# Comparative assessment of empirical and hybrid machine learning models for estimating daily reference evapotranspiration in sub-humid and semi-arid climates

**DOI:** 10.1038/s41598-024-83859-6

**Published:** 2025-01-20

**Authors:** Siham Acharki, Ali Raza, Dinesh Kumar Vishwakarma, Mina Amharref, Abdes Samed Bernoussi, Sudhir Kumar Singh, Nadhir Al-Ansari, Ahmed Z. Dewidar, Ahmed A. Al-Othman, Mohamed A. Mattar

**Affiliations:** 1 Faculty of Sciences and Technologies of Tangier, Abdelmalek Essaadi University, 93000 Tetouan, Morocco; 2 Center for Remote Sensing Applications (CRSA), Mohammed VI Polytechnic University (UM6P), 43150 Benguerir, Morocco; 3 School of Agricultural Engineering, Jiangsu University, Zhenjiang, 212013 People’s Republic of China; 4 Department of Irrigation and Drainage Engineering, G.B. Pant University of Agriculture and Technology, Pantnagar, Uttarakhand 263145 India; 5 K. Banerjee Centre of Atmospheric and Ocean Studies, University of Allahabad, Prayagraj, Uttar Pradesh 211002 India; 6 Department of Civil, Environmental, and Natural Resources Engineering, Lulea University of Technology, 97187 Lulea, Sweden; 7 Prince Sultan Bin Abdulaziz International Prize for Water Chair, Prince Sultan Institute for Environmental, Water and Desert Research, King Saud University, P.O. Box 2454, Riyadh 11451, Saudi Arabia; 8 Department of Agricultural Engineering, College of Food and Agriculture Sciences, King Saud University, Riyadh 11451, Saudi Arabia; 9 Agricultural Engineering Research Institute (AEnRI), Agricultural Research Centre, P.O. Box 256, Giza, Egypt

**Keywords:** Reference evapotranspiration, Light gradient boosting machine, Hybrid model, FAO-56 Penman-Monteith model, Subhumid and semi-arid zones, Agroecology, Agroecology

## Abstract

Improving the accuracy of reference evapotranspiration (RET) estimation is essential for effective water resource management, irrigation planning, and climate change assessments in agricultural systems. The FAO-56 Penman-Monteith (PM-FAO56) model, a widely endorsed approach for RET estimation, often encounters limitations due to the lack of complete meteorological data. This study evaluates the performance of eight empirical models and four machine learning (ML) models, along with their hybrid counterparts, in estimating daily RET within the Gharb and Loukkos irrigated perimeters in Morocco. The ML models examined include Random Forest (RF), M5 Pruned (M5P), eXtreme Gradient Boosting (XGBoost), and Light Gradient Boosting Machine (LightGBM), with hybrid combinations of RF-M5P, RF-XGBoost, RF-LightGBM, and XGBoost-LightGBM. Six input combinations were created, utilizing T_max_, T_min_, RH_mean_, R_s_, and U_2_, with the PM-FAO56 model serving as the benchmark. Model performance was assessed using four statistical indicators: Kling-Gupta efficiency index (KGE), coefficient of determination (R^2^), mean squared error (RMSE), and relative root squared error (RRSE). Results indicate that the Valiantzas 2013 (VAL2013b) model outperformed other empirical models across all stations, achieving high KGE and R^2^ values (0.95–0.97) and low RMSE (0.32–0.35 mm/day) and RRSE (8.14–10.30%). The XGBoost-LightGBM and RF-LightGBM hybrid models exhibited the highest accuracy (average RMSE of 0.015–0.097 mm/day), underscoring the potential of hybrid ML models for RET estimation in subhumid and semi-arid regions, thereby enhancing water resource management and irrigation scheduling.

## Introduction

Reference evapotranspiration (RET) is a crucial hydrological cycle element, responsible for a significant portion of water loss from continental surfaces^1–3^. It accounts for approximately 62% of the rainfall contribution, equivalent to around 73,000 km³ per year^4^. RET serves as a powerful indicator for climate change studies^5–8^ and plays a vital role in many fields, including hydrology, agriculture, ecology, and water resource management. Notably, RET can also be instrumental in addressing natural hazards like dry spells, heat waves, and flash droughts^9–11^. While direct estimation methods like latent heat flux or real evapotranspiration from climate models offer accuracy, they often require complex modelling and high-resolution data, posing practical challenges. The intricate nature of modelling the soil-vegetation-atmosphere interaction further complicates accurate RET estimation. Therefore, understanding the processes and assessing RET is crucial for effective water resource management and planning, especially in semi-arid and dry locations where water availability is restricted. Raza et al.^12^ presented potential evapotranspiration (PET) and RET differentiation and categorized their empirical equations based on different meteorological factors.

The Penman-Monteith technique (PM-FAO56), a revised version of the Penman Equation ^13^, is widely considered as the most accurate method for estimating RET, and has been endorsed by FAO^14^ and the Task Committee on Standardization^15^. Despite its global acceptance, PM-FAO56 relies dependent on meteorological parameters such as wind speed, relative humidity, and solar radiation, which may be unavailable in certain weather stations, particularly in developing countries. Further, this limitation hinders its application in regions with limited weather data. Consequently, researchers have sought alternative methods for estimating RET through comparison of PM-FAO56 method with other empirical methods or methods development through meteorological data obtained from remote sensing.

Numerous researches have been conducted to investigate the effectiveness of different RET methods across different regions and climatic conditions. These studies compare empirical models with PM-FAO56 using different approaches^16–19^, including (a) temperature-based methods, (b) radiation-based methods, (c) mass transfer based methods, (d) methods combining radiation and temperature, and (e) methods integrating radiation, temperature, mass transfer, and other variables. For example, Almorox et al.^18^ assessed eleven temperature-based potential evapotranspiration (PET) estimation methods and determined that the Hargreaves and Samani model exhibited the most accurate performance on a global scale across diverse climatic regions. Additionally, comparisons have been made between empirical models and data obtained from lysimeters^20,21^. In Morocco, few scientists have investigated RET performance with existing empirical models/methods^16,20,22,23^. Er-raki et al.^16^ evaluated three empirical RET estimation methods for Tensift Basin (Morocco’s center) and Yaqui Valley (Northwest Mexico) during 2003–2004. In a semi-arid region, they suggest using Hargreaves and Samani model without calibration (as long as the wind remains low). They suggested that calibration is required for both Priestley-Taylor and Makkink parameters, particularly for dry periods. Several methods were examined by Bouhlassa and Paré^22^ to choose an appropriate solution to PM-FAO56 equation for 1989–2001 in Tafilalet, an arid region in southeastern Morocco. Their findings indicate that Jensen-Haise and Thornthwaite methods best-reflected evapotranspiration obtained by Penman-Monteith-FAO method. Similary, Hadria et al.^23^ conducted a calibration and validation analysis of five temperature-based empirical models in 22 meteorological stations across Morocco. Their results demonstrated that Dorji’s estimate outperformed the other empirical models and they introduced a new fit version called RET-Hadria, specifically designed for assessing RET in arid and semi-arid areas. Zeggaf^20^ compared lysimeter results with various empirical methods for Ouled Gnaou (Morocco’s semi-arid central region) during 1975, 1977, and 1978 years. They found that Priestley-Taylor method gave better results, followed by Penman-Monteith method. Besides, researchers like Liou and Kar^24^ and Elfarkh et al.^25^ have used remote sensing (RS) images and processes in geographical information system (GIS) to enhance evapotranspiration estimation.

In recent years, machine learning models have garnered considerable attention in various fields^26–32^. For instance, a model’s capacity to represent intricate nonlinear relationships has a significant impact on RET estimation. Goyal et al.^33^ highlighted the promising findings of ML models in various climates and environments, emphasizing their ability to improve accuracy above standard empirical models. Besides, researchers have applied various ML models, such as artificial neural networks (ANN)^34,35^, support vector regression (SVR)^36,37^, M5 model tree^38,39^, random forests (RF)^40–43^, reduced error pruning tree (REPTree)^44,45^, extreme gradient boosting (XGBoost)^46–48^, light gradient boosting machine (LightGBM)^34,49,50^ and decision trees (DT)^51–53^ to estimate daily RET uing restricted meteorological data. For instance, Granata^38^ conducted a comparative investigation of M5P Regression Tree, Bagging, RF and SVR with differing input combination (T_mean_, RH_mean_, R_s_, U_2_) for RET estimation in a humid subtropical climate region of Central Florida. They concluded that the M5P models exhibited good performance, while RF proved to be the least accurate. In China, Fan et al.^40^ examined limited meteorological data to investigate four empirical models and three ML models (LightGBM, M5Tree, and RF) to estimate daily RET. Their findings suggested that LightGBM outperformed the other models, with input combinations comprising T_max_, T_min_, U_2_, R_s_, and RH_mean_. Similary, Fan et al.^46^ compared six ML models, including SVM, gradient boosting decision tree (GBDT), M5Tree, XGBoost, ELM and RF, and using meteorological data from eight Chinese stations. Their findings revealed that the GBDT and XGBoost models exhibited performance on par with the SVM and ELM models, while offering advantages in terms of simplicity, accuracy, stability, and reduced computational costs, making them recommended options for daily RET estimation. Additionally, Yong et al.^34^ evaluated performance of LightGBM, ANN and decision forest regression (DFR) in five Malaysian meteorological stations and reported that LightGBM and ANN have proven stable and accurate in determining daily RET. It is worth mentioning that the ML models’ performance in daily RET estimation is influenced by various factors, including the selection of input climatic variables, model structure, basic parameters, and performance criteria. Careful consideration of these factors and the correlation between individual input variables and RET is crucial for optimizing the ML models’ accuracy and efficiency. Additionally, effective tuning of ML model parameters further enhances their performance and efficiency for correct estimation^33–36,38,40,44,46^.

Nowadays, recent studies^33,54^ in RET estimation have emphasized the use of hybrid models, combining multiple ML algorithms through blending or stacking techniques, to address the challenges posed by highly complex meteorological data. Goyal et al.^33^ noted that standalone ML models may not achieve sufficient accuracy in such cases. For example, Elbeltagi et al.^54^ assessed five hybrid models (additive regression (AR) with bagging, M5tree, random subspace, REPTree, ANN, and RF) for a semi-arid area in Pakistan. They concluded that AR-M5tree model is the most appropriate hybrid model for estimating RET. Hence, this highlights the effectiveness of hybrid models in improving performance while maintaining interpretability. However, their RET estimation’s utilization is currently limited, and the available information on this subject is incomplete and fragmented. There is a need for further research and investigation to fully explore the hybrid models’ potential and effectiveness in addressing evapotranspiration estimation challenges.

In Morocco, few studies^51,55,56^ have focused on evaluating ML models for estimating RET. Recently, Lachgar et al.^51^ studied the performance of five ML models, like RF, Linear Regression (LR), SVR, k-Nearest Neighbor (k-NN), and DT for estimating RET between 2011 and 2019 in Fez. They highlighted the ML models’ ability to capture the variance in RET. In Marrakesh, El Hachimi et al.^55^ investigated the performance of SVM, RF, DT, k-NN, LR, XGboost for estimating RET during 2013–2020 and found that XGboost surpasses the other models, followed by RF. To our best knowledge, there is currently no existing comparative research on the use of hybrid models for estimating daily RET in Morocco. Thus, the novelty of this research lies in the comprehensive comparison of empirical models, ML models, and their hybrid models for estimating daily RET in subhumid and semi-arid climates.

The primary objectives of the present research are as follows: (i) estimating RET using eight empirical models, namely, Valiantzas 2013 (VAL2013a and VAL2013b); Dalton 1802 (Dal1802); Trabert 1896 (Trab1896) Hargreaves, 1975 (Harg1975); Irmak and Haman, 2003 (Irs2003); Hargreaves and Samani, 1985 (HargS1985); and Allen and Pruitt, 1986 (BC1986) and comparing their performance with standard FAO-PM56 method, (ii) development of ML models (RF, M5P, XGBoost and LightGBM) and their hybrid models (RF-M5P, RF-XGBoost, RF-LightGBM and XGBoost-LightGBM) using different meteorological input combinations (based on maximum and minimum air temperature (T_max_ and T_min_), relative humidity (RH_mean_), solar radiation (R_s_), and wind speed (U_2_)), (iii) evaluating performance of developed ML and hybrid models using different statistical indices to determine the best for RET estimation. The findings of this research will contribute to assessing the performance and suitability of selected machine learning models for reference evapotranspiration (RET) estimation under the specific climatic conditions of Morocco. This will support improved planning for water resource management and irrigation practices.

## Materials and methods

### Study area and data collection

**Fig. 1:**
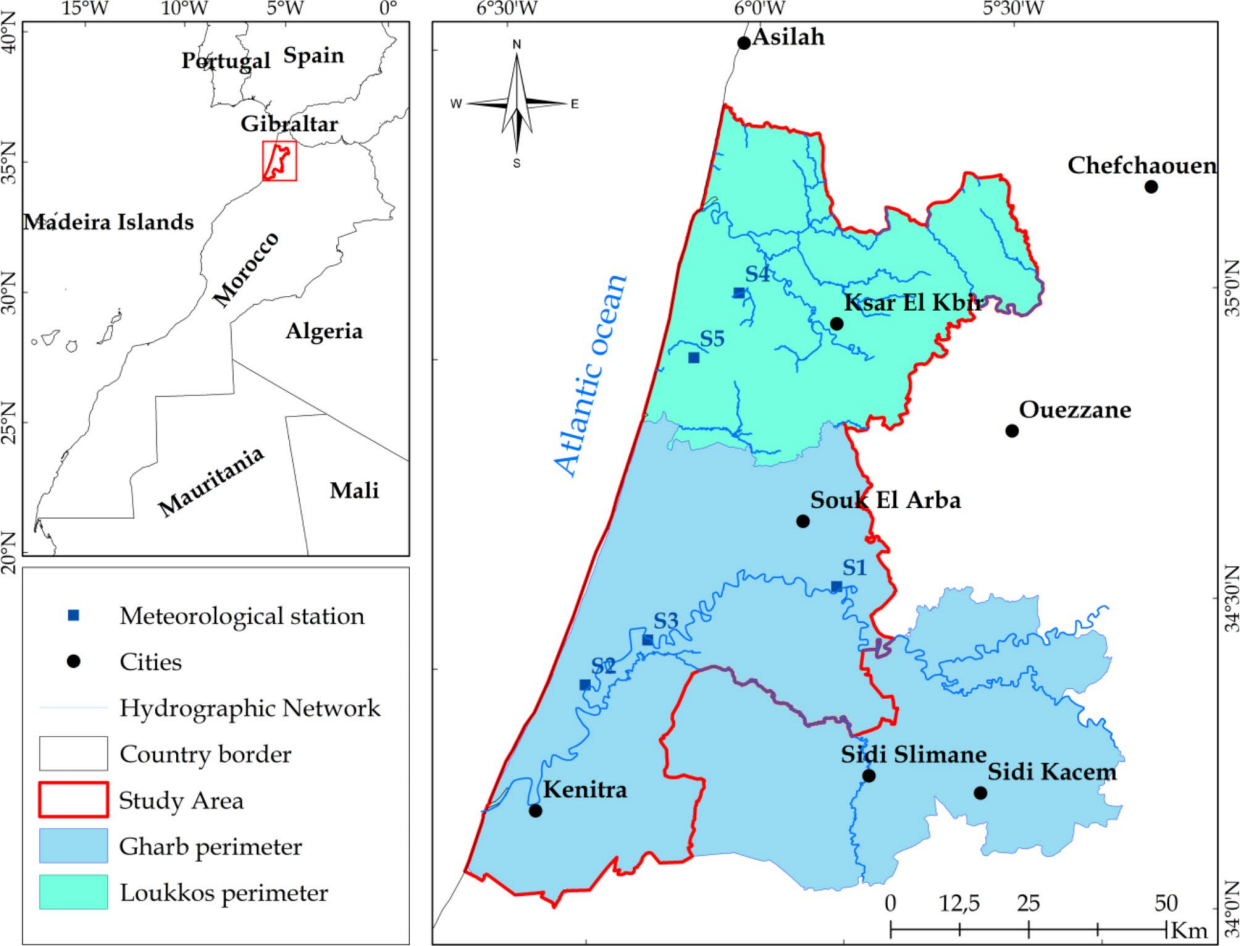
Geographical location of study area, meteorological stations distribution and digital elevation model extracted from Shuttle Radar Topography Mission (SRTM) ) (created by ArcGIS 10.8.2).

Figure 1 illustrates the research region’s geographic distribution. This study region consists of two perimeters which are among Morocco’s most important irrigated perimeters. It covers an area of 6,007.14 km^2^, which is 57.2% for Gharb and 42.8% for Loukkos perimeter. It has a Mediterranean climate (Csa) with an oceanic impact, according to Köppen’s classification. The differences between the two perimeters are shown in Table 1.

**Fig. 2:**
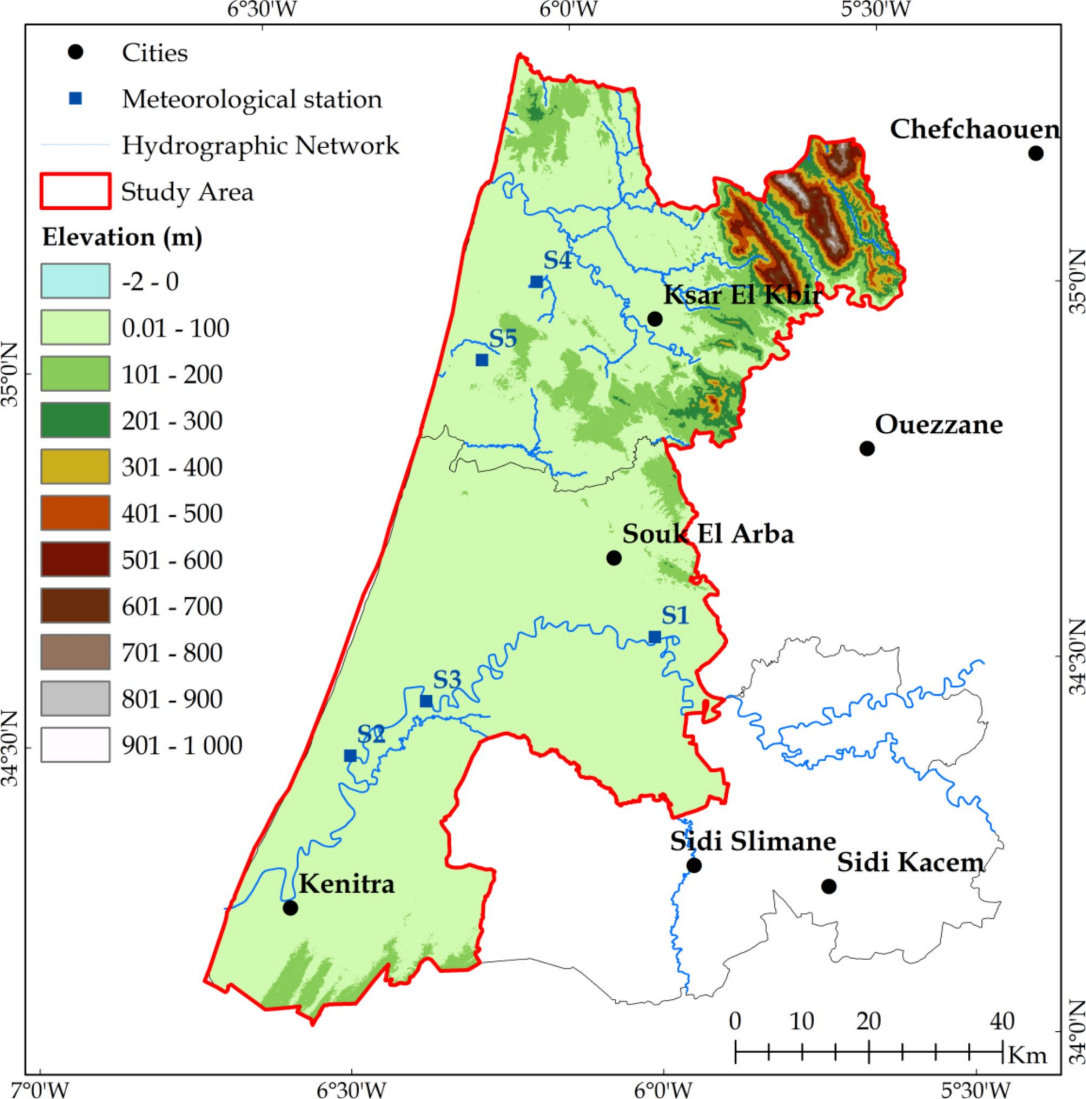
Digital elevation model extracted from Shuttle Radar Topography Mission (SRTM) (created by ArcGIS 10.8.2).

Observed daily minimum and maximum daily air temperature (T_min_ and T_max_), minimum and maximum relative humidity (RH_max_ and RH_min_), solar radiation (R_s_), mean wind speed at 2 m height (U_2_) and precipitation (P) were obtained from five weather stations to estimate reference evapotranspiration. These stations were strategically chosen, with three situated within the Gharb perimeter, representing three out of the five districts, and the remaining two located within the Loukkos perimeter (Figs. 1 and 2). The selection criteria considered the availability of reliable data and practical constraints, allowing for a focused analysis on representative stations within the study area. Meteorological data were provided by two Regional Offices for Agricultural Development, ORMVAG and ORMVAL, with varying data collection periods covering both perimeters. Detailed information on the studied station locations, statistical characteristics of observed meteorological data, and missing data percentage are described in Table 2.

**Table 1:** Difference between two perimeters (Gharb and Loukkos).

Designation	Description	
	Gharb Perimeter	Loukkos Perimeter
Study area	3,435.14 km^2,^ 56% of total perimeter area	2,572 km^2^
Climate type	Subhumid to semi-arid	Subhumid
Number of stations	3	2
Agricultural activity	Intense agricultural activity	Intense agricultural activity
Study period	2011 − 2017	2013 − 2017

**Table 2:** Weather station’s geographical locations and meteorological data annual average values.

	Station	Geographical coordinates			Observations		Measured climatic variables							
		Longitude (°)	Latitude (°)	Altitude (m)	Period	Nbr	T_max_	T_min_	RH_max_	RH_min_	*R*_s_	U_2_	*P*	Prcna
**S1**	Mechraâ Belksiri	5.9	34.6	13.5	P1	670	29.53	13.68	88.9	43.34	0.16	1.18	1.04	4.48
**S2**	Menasra	6.4	34.5	11.5	P2	2192	23.95	12.71	97.61	57.95	0.17	1.61	1.48	3.56
**S3**	Sidi Allal Tazi	6.3	34.5	10.8	P2	2192	25.05	12.54	96.66	55.33	0.17	1.44	1.35	1.37
	Sidi Allal Tazi mice				P2	2192	24.84	12.44	96.75	55.21	0.17	1.43	1.34	1.37*
**S4**	Aouamra	6.1	35.1	28.0	P3	1642	24.62	13.01	95.55	50.68	0.19	2.67	1.5	0
**S5**	SE	6.2	35.0	70.0	P3	1642	22.68	13.14	96.72	57.49	0.19	3.39	2.56	0

With: Nbr: Number of observations. Prcna: Missing data percentage (%). T_max_ and T_min_: Minimum and maximum daily air temperature (°C), respectively. RH_max_ and RH_min_: Minimum and maximum relative humidity (%), respectively. R_s_: Solar radiation (MJ/m^2^/day). U_2_: Mean wind speed at 2m height (m/s). P: Precipitation (mm/day). Period: P1: 1/11/2015 to 31/08/2017, P2: 01/09/2011 to 31/08/2017, P3: 01/01/2013 to 30/06/2017. * reflect that R_S_ missing data is 10.77%.

### Missing data imputation

In general, the database contains missing data due to weather station malfunctions. To address this issue, we employed either deletion or imputation. For the imputation process, we utilized the Multivariate Chain Equations Imputation Method (MICE) developed by Van Buuren and Groothuis-Oudshoorn^57^. This approach enabled us to assess whether imputing missing data would impact the selection of estimation methods. Otherwise, the other missing data was completely removed. The MICE method comprises three main steps. Firstly, a regression model is chosen for the variable being studied. Then, missing data values are iteratively assigned random values based on observed data. Finally, imputed values are estimated using the regression coefficients obtained for each dataset. We opted for this method due to its practicality and effectiveness in analysing precipitation data^58^.

Table 2 reveals that the solar radiation series for the Sidi Allal Tazi station had the highest proportion of missing data, accounting for 10.77% of the observed series. Through the MICE method, we imputed 9.4% of the solar radiation series for the Sidi Allal Tazi station. Overall, the deleted data represented between 1.37% and 4.48% of the database for each station studied.

### Estimating evapotranspiration via FAO-56 Penman-Monteith and empirical models

A series of models were designed by researchers to estimate reference evapotranspiration^59–62^. In this research, eight empirical models, divided into four groups (combination, mass transfer, radiation, and temperature), were selected, as indicated in Table 3. Model selection focused on variables’ availability, usage extent, and simplicity. Subsequently, we compared these models with Penman-Monteith model [PM-FAO56, Eq. 1^14^]. Table 4 summarizes the climate parameters for each empirical model.

**Table 3:** FAO-56 Penman-Monteith and empirical equations used in this research.

Model	Equations		Reference
Target			
FAO-56 Penman-Monteith (PM-FAO56)	$\:{\text{R}\text{E}\text{T}}_{\text{P}\text{M}-\text{F}\text{A}\text{O}56}=$ $\frac{0.408\times\:{\Delta\:}\times\:({\text{R}}_{\text{n}}-\text{G})+{\upgamma\:}\times\:\frac{{\text{C}}_{\text{n}}}{{\text{T}}_{\text{a}\text{v}\text{g}}+273}\times\:({\text{e}}_{\text{s}}-{\text{e}}_{\text{a}}){\text{U}}_{2}}{{\Delta\:}+{\upgamma\:}(1+{\text{C}}_{\text{d}}\times\:{\text{U}}_{2})}\:\:\:$	(1)	Allen et al.^14^
**Mass transfer model**			
Dalton (Dal1802)	$\:{\text{R}\text{E}\text{T}}_{\text{D}\text{a}\text{l}1802}=$ $(3.648+0.7223\times\:{\text{U}}_{2})\times\:({\text{e}}_{\text{s}}-{\text{e}}_{\text{a}})\:\:\:\:$	(2)	Dalton^59^
Trabert (Trab1896)	$\:{\text{R}\text{E}\text{T}}_{\text{T}\text{r}\text{a}\text{b}1896}=$ $(3.075\times\:\sqrt{{\text{U}}_{2}}\times\:({\text{e}}_{\text{s}}-{\text{e}}_{\text{a}})\:\:\:\:$	(3)	Trabert^60^
**Models based on temperature**			
Hargreaves and Samani (HargS1985)	$\:{\text{R}\text{E}\text{T}}_{\text{H}\text{a}\text{r}\text{g}\text{S}1985\text{X}}=$ $0.408\times\:(0.0023\times\:{\text{R}}_{\text{a}}({\text{T}}_{\text{a}\text{v}\text{g}}+17.8)\times\:\sqrt{({\text{T}}_{\text{m}\text{a}\text{x}}-{\text{T}}_{\text{m}\text{i}\text{n}})}$	(4)	Hargreaves^62^
Allen and Pruitt (BC1986)	$\:{\text{R}\text{E}\text{T}}_{\text{B}\text{C}1986}=$ $\text{a}+\text{b}\times\:\text{p}\times\:(0.46{\text{T}}_{\text{a}\text{v}\text{g}}+8.13)(1+0.0001\text{z})\:\:\:\:$	(5)	Allen^63^
**Models based on radiation**			
Hargreaves (Harg1975)	$\:{\text{R}\text{E}\text{T}}_{\text{H}\text{a}\text{r}\text{g}1975}=$ $\left[\right(0.0135\times\:{\text{T}}_{\text{a}\text{v}\text{g}})+0.2403]\times\:\frac{{\text{R}}_{\text{S}}}{{\uplambda\:}}\:\:\:\:$	(6)	Hargreaves^61^
Irmak and Haman (Irs2003)	$\:{\text{R}\text{E}\text{T}}_{\text{I}\text{r}\text{s}2003}=$ $-0.611+0.149\times\:{\text{R}}_{\text{S}}+0.079\times\:{\text{T}}_{\text{a}\text{v}\text{g}}\:\:\:\:$	(7)	Irmak^64^
**Semi-empirical models**			
Valiantzas a (VAL2013a)	$\:{\text{R}\text{E}\text{T}}_{\text{V}\text{A}\text{L}2013\text{a}}=$ $0.393\times\:{\text{R}}_{\text{s}}\times\:\sqrt{({\text{T}}_{\text{a}\text{v}\text{g}}+9.5)}-0.19\times\:({\text{R}}_{\text{s}}{)}^{0.6}\times\:{{\upphi\:}}^{0.15}+0.0061({\text{T}}_{\text{a}\text{v}\text{g}}+20)\times\:[1.12\:{\text{T}}_{\text{a}\text{v}\text{g}}-{\text{T}}_{\text{m}\text{i}\text{n}}-2{]}^{0.7}\:\:\:\:$	(8)	Valiantzas^65^
Valiantzas b (VAL2013b)	$\:{\text{R}\text{E}\text{T}}_{\text{V}\text{A}\text{L}2013\text{b}}=$ $0.393\times\:{\text{R}}_{\text{s}}\times\:\sqrt{({\text{T}}_{\text{a}\text{v}\text{g}}+9.5)}-0.19\times\:({\text{R}}_{\text{s}}{)}^{0.6}\times\:{{\upphi\:}}^{0.15}+0.048\left({\text{T}}_{\text{a}\text{v}\text{g}}+20\right)\left(1-\frac{\text{R}{\text{H}}_{\text{a}\text{v}\text{g}}}{100}\right){{\text{U}}_{2}}^{0.7}\:\:\:\:$	(9)	Valiantzas^65^

RET: Reference evapotranspiration (mm/day). G: Ground heat flux (MJ/m^2^/day). R_n_, R_s_ and R_a_: Net radiation, solar radiation, extra-terrestrial solar radiation respectively (MJ/m^2^/day). γ: Psychometric constant (kPa/°C). e_s_ and e_a_: Saturation vapor pressure and actual vapor pressure respectively (kPa). δ: Saturated vapor pressure/temperature curve slope (kPa/°C). λ: Latent heat of vaporization (MJ/kg). ϕ: Latitude (rad). C_n_: Numerator constant that changes with reference type (K.mm.s^3^/Mg/day). C^d^: Denominator constant that changes with reference type (s/m). Δ: Slope of the saturation vapour pressure curve (kPa/°C). T_min_, T_avg_ and T_max_: Minimum, average and maximum daily air temperature respectively (°C). RH_min_, RH_avg_ and RH_max_: Minimum, average, and maximum relative humidity respectively (%). a, b and p: Regression parameters in BC1986 model. n: Actual duration (h). N: Maximum possible sun or daytime duration (h). P: Precipitations (mm). U_2_: Mean wind speed at 2m (m/s). z: Elevation.

**Table 4:** Climatic parameters required by used models.

Type	Acronym	Parameters		Constants			Variables													
		φ	γ	Z	δ	λ	*R*_*n*_	*R*_s_	*R*_a_	*N*	G	T_avg_	T_max_	T_min_	U_2_	RH_avg_	RH_min_	RH_max_	e_s_	e_a_
Com	PM-FAO56		x		x		x	x			x	x			x				x	x
	VAL2013a	x						x				x		x						
	VAL2013b	x						x				x			x	x				
MT	Dal1802														x				x	x
	Trab1896														x				x	x
Rad	Harg1975					x		x				x								
	Irs2003							x				x								
Tmp	HargS1985								x			x	x	x						
	BC1986			x								x								

Notation: Com: Combination, MT: Mass Transfer, Rad: Radiation and Tmp: Temperature.

### Machine learning models and hybrid models description

Different equations and models to estimate daily RET were compared. Linear Regression (LR) was used to compare eight empirical equations with the PMFAO56 model. Additionally, we explored the impact of various meteorological variables on RET estimation using ML algorithms like Random Forest (RF), M5 Pruned (M5P), eXtreme Gradient Boosting (XGBoost), and Light Gradient Boosting Machine (LightGBM). Additionally, we combined these models to create hybrid models like RF-M5P, RF-XGBoost, RF-LightGBM and XGBoost-LightGBM.

#### Linear regression (LR)

Linear regression is a well-known approach for modelling a dependent variable’s value via one or more independent variables. In this research, The LR equation is written as follows.

10 $$\:{\text{R}\text{E}\text{T}\:}_{\text{c}\text{a}{\text{l}}_{\text{i}}}=\text{a}\times\:{\text{R}\text{E}\text{T}\:}_{\text{P}{\text{M}}_{\text{i}}}+\text{b}$$

$\:{\text{R}\text{E}\text{T}\:}_{\text{P}{\text{M}}_{\text{i}}}$ represents observed values (estimated by PM-FAO 56); $\:{\text{R}\text{E}\text{T}\:}_{\text{c}\text{a}{\text{l}}_{\text{i}}}$ represents values estimated by different empirical models.

#### Random forests (RF)

RF model, introduced by Breiman^66^, is an ensemble approach that integrates numerous decision trees to create a powerful prediction model. It is widely utilized for regression and forecasting tasks due to its ability to capture complex, non-linear interactions between features. It works by generating a collection of random binary trees through bootstrapping, where each tree is trained on a randomly sampled subset of observations from the training dataset. The remaining data, known to as “out-of-bag” (OOB) data, is used for evaluating the model’s performance. RF exhibits several advantages, including strong generalization capabilities, robustness to outliers, and the ability to tune hyperparameters easily. By aggregating the results from individual trees, RF produces a final prediction, often using methods like majority voting. This ensemble approach helps mitigate overfitting and minimize variance by training on various data samples. To further control overfitting, the minimum leaf size parameter can be adjusted, requiring a minimum number of observations to generate child nodes.

#### M5 pruned (M5P)

The M5P model, also known as M5 Pruned, is a decision tree algorithm introduced by Quinlan^67^. This model runs in two steps, providing a novel approach to regression challenges. It separates the input data into subgroups in the first phase and applies linear regression models to each subset based on their partial attribute values. This enables the model to record variable relationships and construct regression equations at each node. Furthermore, the M5P model is typically built as a tree, starting with a root node and branching out into subnodes that reflect the regression equations. It can easily handle huge datasets with high dimensionality^68^, making it useful for investigating complicated systems like evapotranspiration estimates. Additionally, it does not require parameter adjustment, which simplifies the process.

#### Extreme gradient boosting (XGBoost)

XGBoost model is an improved Gradient Boosting Machines (GBMs) version presented by Chen and Guestrin^69^ that expands on the notion of “boosting” weak learners. Through additive training procedures, it combines numerous weak models to generate a powerful learner. By simplifying goal functions and providing parallel calculations during training, XGBoost tries to minimize overfitting while decreasing computational costs. It offers a scalable and effective solution for both regression and classification workloads^27^. With features such as distributed computing, pruning strategies, and management of missing data, the approach is meant to maximize speed. Because of its efficacy, versatility, and capacity to handle big datasets, XGBoost has become a popular choice.

#### Light gradient boosting machine (LightGBM)

LightGBM model is a gradient-boosting architecture that improves model performance while using less memory than conventional models. LightGBM is distinguished by its novel leaf-wise development, which develops trees by focusing on individual leaves rather than sequentially expanding the branches^70^. This approach enables efficient tree formation and increased computing performance. LightGBM also incorporates two approaches: gradient-based one-sided sampling and exclusive feature bundling (EFB). These approaches improve model performance by allowing for more efficient feature sampling and grouping. Overall, LightGBM is an efficient technique for dealing with massive datasets and producing accurate predictions while conserving resources. One of the most essential variables impacting the accuracy of a given model is the selection of proper hyper-parameters. The hyper-parameters employed in this study, shown in Table 5, were chosen through grid search optimization, supported by prior domain knowledge. This approach enabled systematic evaluation of parameter values to minimize prediction error and optimize performance. The resulting tuning achieved a balance between computational efficiency and accuracy across all models.

**Table 5:** ML algorithm hyper-parameters.

ML Model name	Parameters description
LR	Attribute Selection Method = M5 method, Batch size-100, Debug = False, Eliminate Collinear Attribute = True, Minimal = False
Random Forests (RF)	Batch size- 100, bag Size percent = 100, max depth = 0, numbers of executions slots = 1, number of iterations = 100, and random seed = 1
M5 Pruned (M5P)	Batch size-100, Minimum number of instances = 4
Extreme gradient boosting (XGBoost)	Estimator number = 1000, Max depth = 5, Learning rate: 0.1
Light gradient boosting machine (LightGBM)	Estimator number = 1000, Max depth = 5, Learning rate: 0.1

### Weighted hybridization for ML algorithms

Weighted hybridization is an approach that combines different algorithms to increase the accuracy of evapotranspiration estimate. Individual algorithms are given varied weights based on their performance, as proposed by Nourani et al.^36^. More information can be found in^33,71^. The hybrid model provides more accurate and resilient predictions by harnessing the capabilities of each algorithm and assessing their relative relevance. This method is critical for reducing the constraints of individual algorithms and increasing the dependability of evapotranspiration estimations.

To ensure consistent scale and improve modelling capabilities, the input data for the ML models were normalised. This process, described by Eq. (11), transformed the data to a range between 0 and 1.

11 $$\:{x}_{norm}=\frac{{x}_{0}-{x}_{min}}{{x}_{max}-{x}_{min}}$$

where, x_norm_ represents the normalised value, x_0_ is the real value, and x_min_ and x_max_ are the minimum and maximum values respectively.

It should be noted that the XGBoost model does not require input variable normalization since it is not sensitive to monotonic input variable normalization.

### Input combinations

To investigate the impact of different meteorological variables on RET estimation, six combinations were utilized, as outlined in Table 6. The inputs for the ML models included air temperature (T_max_ and T_min_), relative humidity (RH_mean_), solar radiation (R_s_), and wind speed (U_2_). Likewise, the observed meteorological data were divided into two sets: a training set comprising 70% of the data and a separate testing set for evaluating the performance of the models. This division ensured that the models thoroughly evaluated on a substantial amount of data from each location and were rigorously assessed on an independent dataset. All the chosen ML models and simulations were implemented using R software (version 4.2.2).

**Table 6:** ML models, hybrid models and input meteorological combinations.

No	Models								Input combinations	
	RF	M5P	XGBoost	LightGBM	RF-M5P	RF-LightGBM	RF-XGBoost	XGBoost-LightGBM		
1	RF1	M5P1	XGBoost1	LightGBM1	RF-M5P1	RF-LightGBM1	RF-XGBoost1	XGBoost-LightGBM1	Comb. 1	T_max_, T_min_, R_s_
2	RF2	M5P2	XGBoost2	LightGBM2	RF-M5P2	RF-LightGBM2	RF-XGBoost2	XGBoost-LightGBM2	Comb. 2	T_max_, T_min_, RH_mean_
3	RF3	M5P3	XGBoost3	LightGBM3	RF-M5P3	RF-LightGBM3	RF-XGBoost3	XGBoost-LightGBM3	Comb. 3	T_max_, T_min_, R_s_, U_2_
4	RF4	M5P4	XGBoost4	LightGBM4	RF-M5P4	RF-LightGBM4	RF-XGBoost4	XGBoost-LightGBM4	Comb. 4	T_max_, T_min_, RH_mean_, U_2_
5	RF5	M5P5	XGBoost5	LightGBM5	RF-M5P5	RF-LightGBM5	RF-XGBoost5	XGBoost-LightGBM5	Comb. 5	T_max_, T_min_, RH_mean_, R_s_
6	RF6	M5P6	XGBoost6	LightGBM6	RF-M5P6	RF-LightGBM6	RF-XGBoost6	XGBoost-LightGBM6	Comb. 6	T_max_, T_min_, RH_mean_, R_s_, U_2_

### Evaluation performance

To evaluate the model’s performance in comparison to the standard Penman-Monteith model (FAO-PM56), four statistical metric parameters were employed. These metrics encompassed the Kling Gupta Efficiency index (KGE, Eq. 12), Coefficient of determination (R^2^, Eq. 13), Mean Squared Error (RMSE, Eq. 14), and Root relative squared error (RRSE, Eq. 15). The selection of these metrics aimed to assess the precision, accuracy, under/overestimation, and provide a means for model comparison^72–74^. To rank the models, the RMSE and RRSE values were arranged in ascending order, while the R^2^ and KGE values were ordered in descending order. The specific formulas for these metrics can be found in Table 7.

**Table 7:** Statistical metrics parameters.

Metric parameter	Equation	
Kling Gupta Efficiency index	$\:\text{K}\text{G}\text{E}=1-\text{E}\text{D}$ $\:\text{W}\text{i}\text{t}\text{h}\:\text{E}\text{D}=[{\text{s}}_{\text{r}}\times\:(\text{r}-1){]}^{2}+[{\text{s}}_{{\upalpha\:}}\times\:({\upalpha\:}-1){]}^{2}+[{\text{s}}_{{\upbeta\:}}\times\:({\upbeta\:}-1){]}^{2}$	(12)
Coefficient of determination	$\:{\text{R}}^{2}=\frac{\sum\:_{\text{i}=1}^{\text{N}}\, \left[\right({\text{R}\text{E}\text{T}\:}_{\text{P}{\text{M}}_{\text{i}}}-\overline{{\text{R}\text{E}\text{T}\:}_{\text{P}{\text{M}}_{\text{i}}}})\times\:({\text{R}\text{E}\text{T}\:}_{\text{c}\text{a}{\text{l}}_{\text{i}}}-\overline{{\text{R}\text{E}\text{T}\:}_{\text{c}\text{a}{\text{l}}_{\text{i}}}}){]}^{2}}{\sum\:_{\text{i}=1}^{\text{N}}\, ({\text{R}\text{E}\text{T}\:}_{\text{P}{\text{M}}_{\text{i}}}-\overline{{\text{R}\text{E}\text{T}\:}_{\text{P}{\text{M}}_{\text{i}}}})\times\:({\text{R}\text{E}\text{T}\:}_{\text{c}\text{a}{\text{l}}_{\text{i}}}-\overline{{\text{R}\text{E}\text{T}\:}_{\text{c}\text{a}{\text{l}}_{\text{i}}}})}$	(13)
Mean Squared Error	$\:\text{R}\text{M}\text{S}\text{E}=\sqrt{\frac{1}{\text{N}}\times\:\sum\:_{\text{i}=1}^{\text{N}}\, ({\text{R}\text{E}\text{T}\:}_{\text{P}{\text{M}}_{\text{i}}}-\:{\text{R}\text{E}\text{T}\:}_{\text{c}\text{a}{\text{l}}_{\text{i}}}{)}^{2}}$	(14)
Root relative squared error	$\:RRSE=\frac{\sqrt{\sum\:_{i=1}^{N}{\left({\text{R}\text{E}\text{T}\:}_{\text{c}\text{a}{\text{l}}_{\text{i}}}-{\text{R}\text{E}\text{T}\:}_{\text{P}{\text{M}}_{\text{i}}}\right)}^{2}}}{\sqrt{\sum\:_{i=1}^{N}{\left({\text{R}\text{E}\text{T}\:}_{\text{P}{\text{M}}_{\text{i}}}-{\text{R}\text{E}\text{T}\:}_{\text{c}\text{a}{\text{l}}_{\text{i}}}\right)}^{2}}}$	(15)

N: is the total number of observations; k is the number of parameters; ED is Euclidean distance from the ideal point in scale space; r is the correlation coefficient; $\:{\upalpha\:}$ is representing the variability of prediction errors, β is the ratio between calculated RET and RET PM-FAO 56, which means that β is a bias; $\:{\text{s}}_{\text{r}}$, $\:{\text{s}}_{{\upalpha\:}}\:$and $\:{\text{s}}_{{\upbeta\:}}$ are scale factors that can be applied to reduce criteria space before calculating Euclidean distance from the ideal point. In other words, $\:{\text{s}}_{\text{r}}$, $\:{\text{s}}_{{\upalpha\:}}\:$and $\:{\text{s}}_{{\upbeta\:}}$ can be used to adjust emphasis on different components. $\:\overline{{\text{R}\text{E}\text{T}\:}_{\text{P}{\text{M}}_{\text{i}}}}$ and $\:\overline{{\text{R}\text{E}\text{T}\:}_{\text{c}\text{a}{\text{l}}_{\text{i}}}}$ are respectively average values of RET$\:{\:}_{\text{P}{\text{M}}_{\text{i}}}$ and RET$\:{\:}_{\text{c}\text{a}{\text{l}}_{\text{i}}}$.

### Proposed model development for RET estimation

**Fig. 3:**
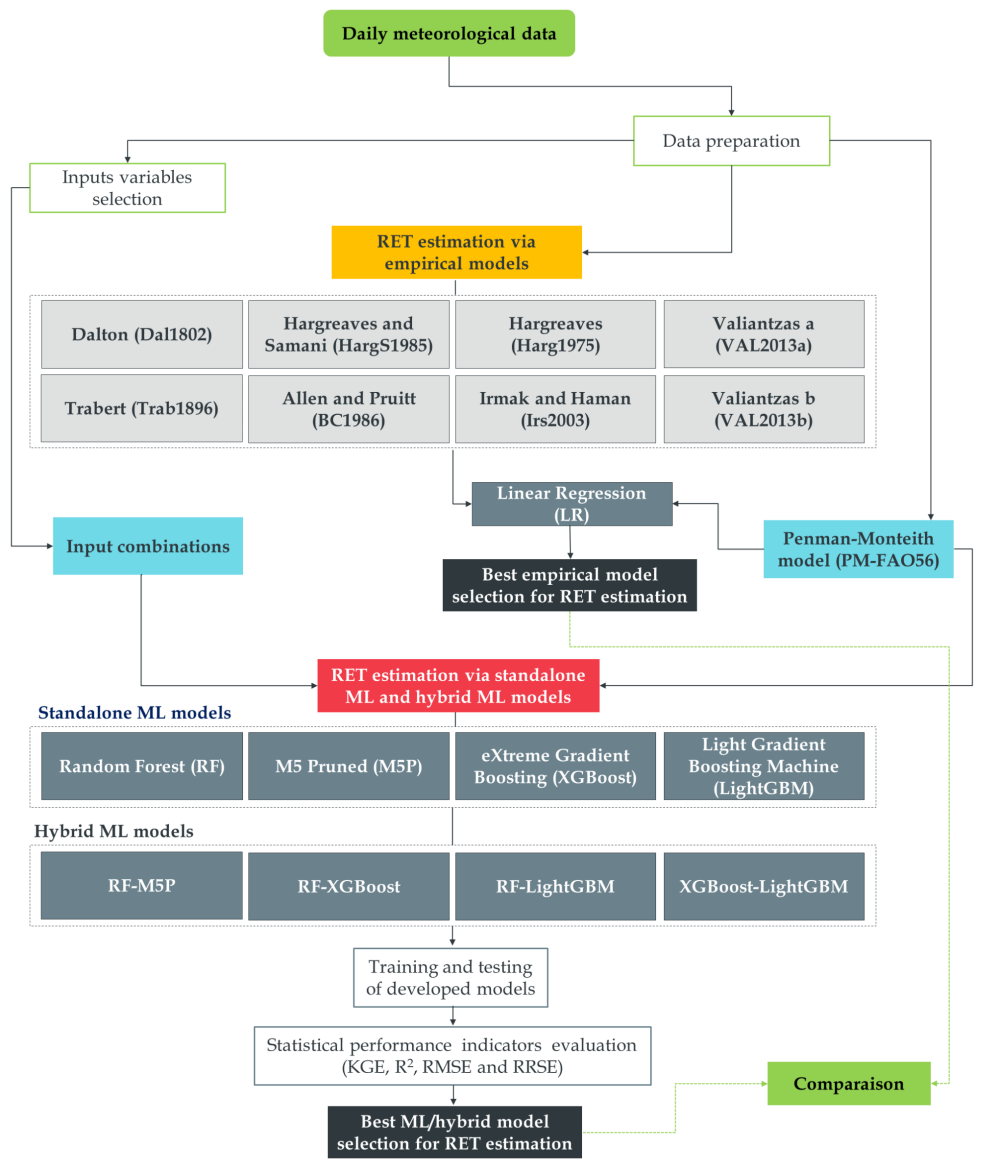
Proposed empirical, ML and hybrid models flowchart for RET estimation.

Figure 3 illustrates the flowchart outlining the suggested empirical, machine learning (ML), and hybrid models for estimating reference evapotranspiration (RET). LR, RF, M5P, XGBoost, LightGBM, RF-M5P, RF-XGBoost, RF-LightGBM, and XGBoost-LightGBM are the models used for RET estimation. As input variables, these models use six distinct climatic combinations (Comb1 - Comb6). The performance of each model was extensively explored by assessing the statistical metrics parameters listed above.

## Results and discussions

### Correlation between PM-FAO 56 daily RET and meteorological variables

**Fig. 4:**
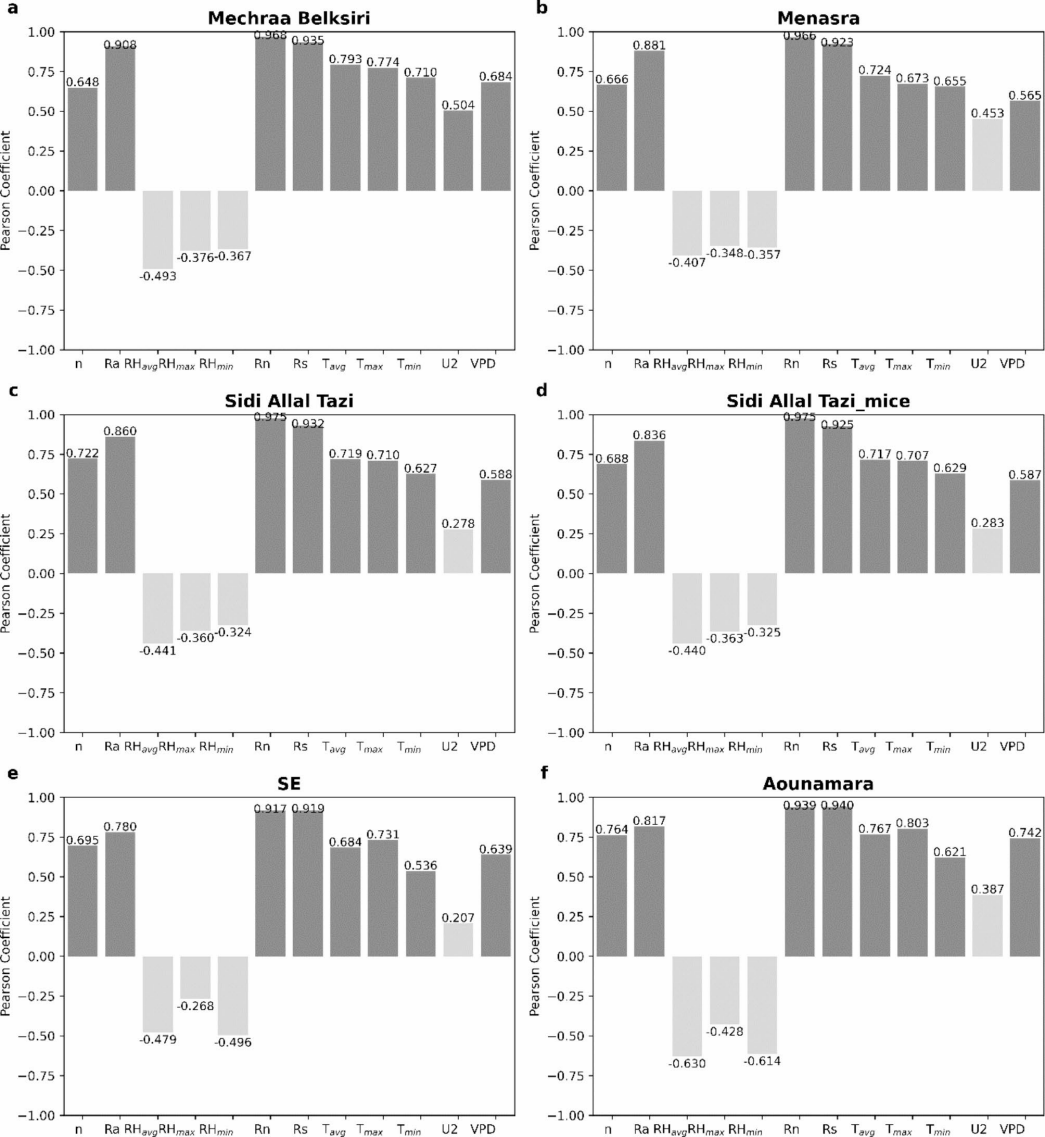
Correlation of Pearson coefficients between daily RET calculated by PM-FAO 56 model and meteorological variables for different weather stations.

Figure 4 shows a correlation between meteorological variables and daily reference evapotranspiration estimated by PM-FAO 56 model at each station studied. Results shows the RET is primarily affected by solar radiation where Pearson’s coefficients were above 78%, indicating a good correlation. This suggests that models that incorporate radiation-based variables may perform better in estimating RET compared to those that rely on temperature-based or mass transfer-based variables. For all Gharb stations, R_n_ obtained the highest correlation, with values ranging from 0.96 to 0.97, followed by R_s_ and R_a_. However, R_s_ is the most correlated to R_n_ and R_a_ for all Loukkos stations. One of these three variables is explicitly contained in all combination models and those based on radiation-based (Table 3). Temperatures occupy the second place as r values vary from 0.53 to 0.79. Moreover, maximum temperature for Loukkos stations is more correlated with reference evapotranspiration than with mean and minimal temperatures. This leads to saying that a model containing maximum temperature coupled with solar radiation could better estimate RET for these stations. Recent research by Chia et al.^71^ pointed out that temperature and radiation are indispensable for estimating RET in semi-arid regions. Conversely, in sub-humid regions, the RET estimation requires the inclusion of evaporation in addition to temperature and radiation. This review underscores the significance of considering distinct climatic conditions when estimating RET in various regions.

Figure 4 further indicate that air vapor pressure deficit (VPD) is moderately correlated with reference evapotranspiration ranging from 0.56 to 0.74. Moreover, mass transfer models usually use this VPD variable as input and lack other variables that could improve RET estimation. On the other hand, relative humidity is negatively correlated with reference evapotranspiration, with r values varying from − 0.63 to -0.27. In the Gharb stations, it is noteworthy that RH_max_ correlation coefficient is higher than RH_min_, regardless of the study period. In line with previous studies^33,44,71^, our findings support the positive correlation of T_mean_, T_max_, T_min_, and R_s_ with RET. Wind speed shows a slight correlation, while relative humidity (RH_mean_) exhibits a negative correlation with RET. The correlation coefficients for wind speed (U_2_) are relatively low, ranging from 0.21 to 0.40, with the exception of the MB station, which exhibits a correlation coefficient of 0.50. The consideration of wind impact in RET estimation varies among researchers, with some arguing that wind is a significant factor due to potential data inaccuracies^75^, while others suggest that wind has minimal influence^76^ except in areas with high wind conditions. These results align with the findings of other studies^46,54^, providing further evidence of the relationship between meteorological variables and RET.

### Empirical models’ comparison for daily reference evapotranspiration estimates

The statistical results of the eight empirical models (Dal1802, Trab1896, Harg1975, Irs2003, HargS1985, BC1986, VAL2013a and VAL2013b) for estimating daily RET at the five meteorological stations within the Gharb and Loukkos perimeters are presented in Tables 8 and 9. As above mentioned, the computed statistical indicator values (KGE, R^2^, RMSE and RRSE) were obtained using model performance equations [Eqs. 12–15], evaluated against the PM - FAO 56 model during the training and testing phases. The models were prioritized based on their statistical errors, and the best model was identified accordingly. KGE, R^2^, RMSE, and RRSE were determined to be 0.291–0.974, 0.249–0.964, 0.182–1.287 mm/day and 8.572–51.913%, respectively, during training; and 0.386–0.972, 0.366–0.966, 0.172–1.302 mm/day and 8.137–47.993% during testing. As seen in the table, the empirical models exhibited minimal differences (with an RRSE gap from − 1.57 to 4.63%) between the training and testing phases.

Notably, the combination models (VAL2013a, VAL2013b) outperformed other models across all stations, with KGE R^2^, RMSE, and RRSE ranging 0.947–0.974, 0.942–0.966, 0.318–0.402, 8.137–10.739 respectively, during testing phase. Except for Mensara station, Trab1896 performed successfully at the training and testing phases. It was noticed that The VAL13b model performs noticeably better than the VAL13a model, owing to the distinct variable requirements of each model. This is because it includes all contributing factors affecting RET.

**Table 8:** Statistical indicators result for evaluating empirical model performance in Gharb perimeter.

Station			Model	Training					Testing				
				KGE	R^2^	RMSE	RRSE	Rank	KGE	R^2^	RMSE	RRSE	Rank
Gharb	Mechrâa Belksiri (MB)	MT	Dal1802	0.618	0.532	1.161	31.629	8	0.575	0.543	1.174	31.641	8
			Trab1896	0.654	0.570	1.113	30.329	7	0.629	0.588	1.112	29.950	7
		Tmp	HargS1985	**0.966**	**0.953**	**0.368**	**10.024**	1	0.962	0.949	0.393	10.580	2
			BC1986	0.871	0.826	0.709	19.322	6	0.840	0.847	0.685	18.463	5
		Rad	Harg1975	0.958	0.942	0.411	11.184	3	0.955	0.936	0.437	11.766	3
			Irs2003	0.876	0.860	0.680	17.800	5	0.846	0.840	0.660	18.200	6
		Com	VAL2013a	0.936	0.912	0.505	13.742	4	0.924	0.910	0.520	14.021	4
			VAL2013b	0.961	0.946	0.394	10.739	2	**0.965**	**0.951**	**0.382**	**10.301**	1
	Menasra (M)	MT	Dal1802	0.434	0.359	0.316	47.945	4	0.510	0.470	0.291	44.286	4
			Trab1896	**0.841**	**0.788**	**0.182**	**27.593**	1	**0.878**	**0.815**	**0.172**	**26.209**	1
		Tmp	HargS1985	0.360	0.300	0.330	50.134	6	0.418	0.391	0.312	47.512	6
			BC1986	0.291	0.249	0.342	51.913	8	0.386	0.383	0.316	47.993	7
		Rad	Harg1975	0.516	0.433	0.297	45.105	3	0.587	0.533	0.273	41.508	3
			Irs2003	0.380	0.316	0.326	49.551	5	0.437	0.377	0.315	47.902	8
		Com	VAL2013a	0.641	0.557	0.263	39.879	2	0.736	0.662	0.232	35.249	2
			VAL2013b	0.358	0.298	0.330	50.188	7	0.452	0.432	0.302	45.977	5
	Sidi Allal Tazi (SAT)	MT	Dal1802	0.470	0.391	1.284	36.308	8	0.493	0.366	1.302	37.621	8
			Trab1896	0.497	0.415	1.258	35.573	7	0.517	0.399	1.263	36.492	7
		Tmp	HargS1985	0.968	0.955	0.349	9.862	2	0.962	0.956	0.340	9.809	2
			BC1986	0.803	0.741	0.837	23.657	6	0.820	0.729	0.853	24.641	6
		Rad	Harg1975	0.966	0.953	0.357	10.100	3	0.964	0.953	0.350	10.111	3
			Irs2003	0.898	0.861	0.614	17.363	5	0.887	0.842	0.645	18.637	5
		Com	VAL2013a	0.961	0.946	0.383	10.831	4	0.959	0.942	0.390	11.269	4
			VAL2013b	**0.973**	**0.963**	**0.318**	**8.992**	1	**0.972**	**0.961**	**0.320**	**9.259**	1
	Sidi Allal Tazi mice (SATmice)	MT	Dal1802	0.462	0.384	1.265	35.917	8	0.482	0.373	1.292	37.462	8
			Trab1896	0.494	0.412	1.236	35.088	7	0.513	0.399	1.264	36.656	7
		Tmp	HargS1985	0.962	0.947	0.371	10.535	2	0.952	0.943	0.388	11.254	2
			BC1986	0.786	0.720	0.853	24.223	6	0.809	0.734	0.839	24.337	6
		Rad	Harg1975	0.960	0.944	0.381	10.831	3	0.953	0.942	0.393	11.397	3
			Irs2003	0.887	0.847	0.631	17.908	5	0.885	0.854	0.621	18.019	5
		Com	VAL2013a	0.955	0.937	0.404	11.459	4	0.948	0.933	0.423	12.272	4
			VAL2013b	**0.968**	**0.955**	**0.340**	**9.655**	1	**0.961**	**0.954**	**0.348**	**10.083**	1

Significant values are in bold.

**Table 9:** Statistical indicators result for evaluating empirical model performance in Loukkos perimeter.

Station			Model	Training					Testing				
				KGE	R^2^	RMSE	RRSE	Rank	KGE	R^2^	RMSE	RRSE	Rank
Loukkos	Aouamra (Aou)		Dal1802	0.655	0.571	1.213	29.378	8	0.676	0.561	1.218	29.366	8
		MT	Trab1896	0.663	0.580	1.202	29.095	7	0.681	0.563	1.215	29.300	7
			HargS1985	0.964	0.950	0.416	10.076	2	0.958	0.939	0.454	10.945	3
		Tmp	BC1986	0.938	0.914	0.545	13.186	5	0.952	0.911	0.550	13.271	5
			Harg1975	0.952	0.933	0.481	11.647	4	0.946	0.921	0.516	12.445	4
		Rad	Irs2003	0.921	0.891	0.611	14.803	6	0.918	0.905	0.567	13.679	6
			VAL2013a	0.961	0.946	0.432	10.453	3	0.959	0.942	0.443	10.687	2
		Com	VAL2013b	**0.974**	**0.964**	**0.354**	**8.572**	1	**0.967**	**0.966**	**0.337**	**8.137**	1
	SE	MT	Dal1802	0.490	0.409	1.287	32.806	8	0.449	0.484	1.249	31.521	8
			Trab1896	0.498	0.416	1.279	32.607	7	0.456	0.491	1.240	31.306	7
		Tmp	HargS1985	0.937	0.913	0.493	12.560	3	0.948	0.928	0.456	11.500	3
			BC1986	0.899	0.862	0.622	15.854	5	0.910	0.869	0.614	15.489	6
		Rad	Harg1975	0.926	0.898	0.535	13.627	4	0.938	0.913	0.500	12.634	4
			Irs2003	0.882	0.840	0.670	17.079	6	0.876	0.879	0.595	15.023	5
		Com	VAL2013a	0.944	0.923	0.465	11.851	2	0.955	0.936	0.429	10.842	2
			VAL2013b	**0.959**	**0.942**	**0.402**	**10.248**	1	**0.947**	**0.960**	**0.343**	**8.672**	1

Significant values are in bold.

Notably, VAL13b incorporates additional variables, such as relative humidity and wind speed, which account for the aerodynamic effects on RET. Similary, Kisi^77^ evaluated seven empirical models to the PM-FAO 56 in Mediterranean climate and Valiantzas (2013b) was found to be the best model.

The analysis indicates that temperature-based models, specifically Hargreaves and Samani^62^ and Brutsaert and Chen^78^, demonstrate superior performance compared to radiation-based models such as Hargreaves^61^ and Irmak et al.^79^, as well as mass transfer models like Dalton^59^ and Trabert^60^. These findings are consistent with the conclusions reported by multiple researchers in the field^17^. Conversely, it is important to note that some researchers^20,76,80^ have suggested that radiation-based models may perform better than temperature-based models. The higher effectiveness of temperature-based models can be related to temperature’s relative stability compared to solar radiation, which changes depending on conditions like as cloud cover, meteorological conditions, and time of day. Consequently, variations in solar radiation create uncertainty in radiation-based models. Generally, temperature appears to be a more powerful element than solar radiation in promoting evapotranspiration in dry or semi-arid regions where water supply is limited^72–74^.

Specifically, the HargS1985 model demonstrates superior performance compared to the BC1986 model across all stations. This finding is congruent with Er-raki et al.^16^ results, who found that the HargS1985 model provides more accurate estimations in semi-arid conditions of the Tensift basin. Similarly, Almorox et al.^18^ reported that the HargS1985 model had the greatest overall performance after analyzing eleven temperature-based models on a worldwide scale. In contrast, other studies have reported that the Hargreaves-Samani model^62^ tends to underestimate reference evapotranspiration in arid regions and overestimate it in wetland environments^81,82^.

In term of mass transfer models, Trab1896 performed better than Dal1802 at most stations. The mass transfer models used in this study were unsuitable for estimating daily RET in this region due to substantial statistical errors, with the exception of the Menasra station. In term of radiation models, Harg1975 was generally superior to Irs2003. On the other hand, we found that RET ranking is generally like SAT station data and data supplemented by Mice imputation method (SAT mice). This means that the filling of solar radiation (R_s_) gaps did not affect the choice of RET estimation method.

**Fig. 5:**
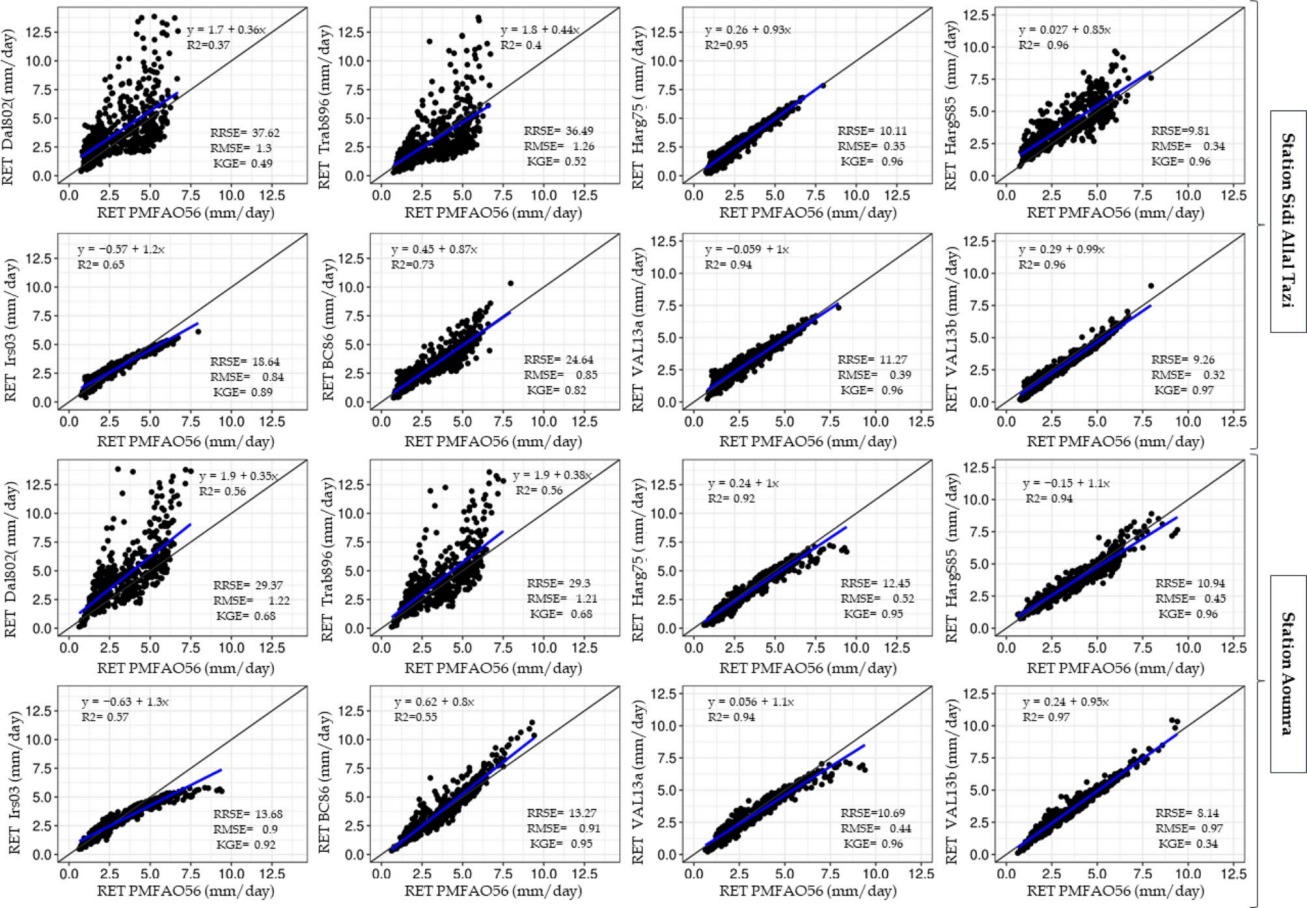
Scatter plots depicting the relationship between RET values estimated by different empirical models and FAO-56 PM values during testing phase at Sidi Allal Tazi and Aouamra stations.

The scatter plots in Fig. 5 illustrate the comparison between the estimated RET values obtained from the best empirical models and the FAO56-PM values during testing at the two meteorological stations. When the data points in the scatter plot were closely matched with the diagonal 1:1 trend line, a strong fit was noticed, showing high agreement between RET estimated by PM-FAO 56 and by empirical model. When the data points deviated considerably from the trend line, it indicated a poor fit, suggesting a lack of connectivity between the two previously estimated models. Overall, the data points in the plots demonstrate a strong correlation, aligning closely to the 1:1 line for models Val2013a, Val2013b, Harg195, and Harg1985.

### Comparison of standalone and hybrid ML models using various input combinations

**Fig. 6:**
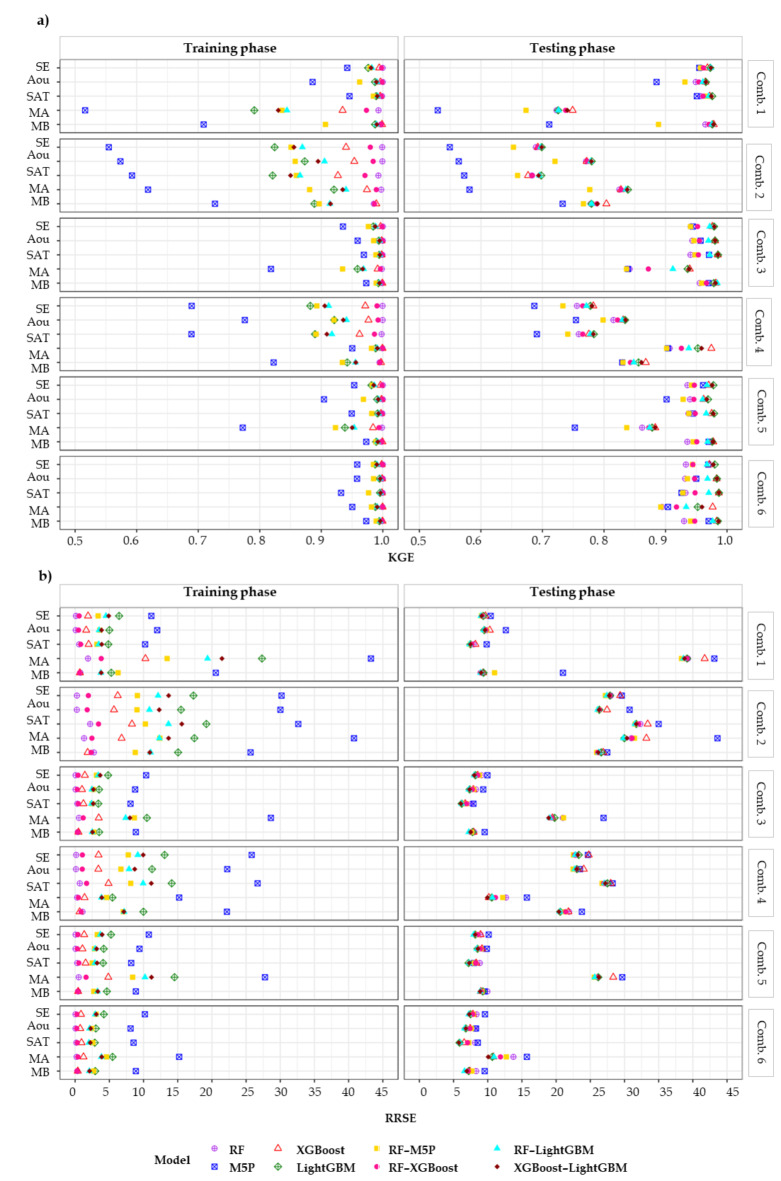
KGE and RRSE results for each meteorological station throughout training and testing.

Table 10 presents the averaged statistical performance indicators (KGE, R^2^, RMSE, and RRSE) values for estimating RET across five meteorological stations in the Loukkos and Gharb perimeters, categorized by model and input combination used during testing phase. Additionally, Fig. 6 displays the KGE and RRSE values for each meteorological station during training and testing phases, comparing the four ML models and four hybrid models with six different input combinations. Overall, the statistical indicators values were found to varied substantially based on input combination, model types, and phase employed.

It can be seen from Table 10 that, independent of the perimeter and input combination, KGE, R^2^, RMSE, and RRSE values varied from 0.557 to 0.982, 0.585–0.979, 0.015–0.108, and 6.925–35.360 respectively during testing phase. These results clearly surpass those produced by the empirical model. The RMSE values achieved by these models were smaller than those reported by other researchers using different ML models in various regions^34,40,46,51^. It’s worth noting that discrepancies in RMSE values between studies might be caused by various factors such as ML model type and input variable selection, time periods chosen, climate conditions, and data quality^83–85^.

Among the different input combinations evaluated across all eight models, it was observed that the ML and hybrid models displayed the poorest performance when utilizing T_max_, T_min_, and RH_mean_ as input (combination 2). On average, the R^2^ values ranged from 0.585 to 0.669, while the RRSE values varied between 26.724% and 35.360%, indicating relatively lower accuracy compared to other input combinations. This can be attributed to the negative correlation observed between RH_mean_ and RET estimated, as depicted in Fig. 4. Furthermore, the limited information provided by these variables may not fully capture the complex relationships involved in accurately estimating RET. When comparing combination 1 (T_max_, T_min_, R_s_) with combination 3 (T_max_, T_min_, R_s_, U_2_), it was observed that the inclusion of U_2_ improve slightly the R^2^ (difference < 0.12) and reduced RMSE values (difference < 0.01). However, the most significant difference was found in the RRSE values, particularly in the Gharb stations, where the improvements ranged from 6.597 to 9.862%.

In the Loukkos stations, the differences in RRSE values were between 0.910% and 1.886%. These disparities suggest that the Loukkos stations experience higher wind speeds compared to those in the Gharb (Table 2), and the correlation between U_2_ and RET is slightly weaker in the Loukkos (as shown in Fig. 4). Similarly, when comparing combination 1 (T_max_, T_min_, R_s_) with combination 5 (T_max_, T_min_, R_s_, RH_mean_), improvements were observed, although they were lower than in the previous case. The RRSE differences were 3.871–8.923% for Gharb and 0.499–1.537% for Loukkos.

Goyal et al.^33^ suggested that incorporating either U_2_, RH_mean_, or both can improve model performance. Although relative humidity is considered the least significant parameter, its addition to the combination of T_max_, T_min_, and R_s_ results in a decrease in RMSE values. Besides, the combination 6, which included T_max_, T_min_, RH_mean_, R_s_, and U_2_ as input meteorological variables, demonstrated the best performance with R^2^ and RRSE values ranging from 0.955 to 0.979 and from 6.925 to 11.272%, respectively. This improved performance can be attributed to the inclusion of additional variables capture the complex interactions and dynamics involved in estimating RET accurately.

Overall, it’s worth noting that temperature data is a foundational requirement for the models presented in Tables 9 and 10. Without sufficient temperature data, model predictions become unreliable, thereby limiting accuracy of RET estimations. Alternative gridded datasets, such as those from reanalysis products like ERA5-Land, can be used effectively to run models like Penman-Monteith FAO-56^86^. Nouri et al.^86^ demonstrated that the ERA5-Land dataset provides reliable RET estimates, especially in data-limited and windy regions. Their findings revealed that while some models, like recalibrated Hargreaves-Samani and Penman-Monteith with localized wind speed, performed well, others struggled due to wind speed variation.

In term of standalone ML models, the testing phase revealed the following ranking for Gharb: LightGBM6 > XGBoost6 > RF6 > LightGBM3, with average RMSE of 0.015–0.017 mm/day. For Loukkos, the ranking was: LightGBM6 > XGBoost6 > LightGBM3 > XGBoost3 with average RMSE of 0.025–0.027 mm/day. These findings support previous studies by Fan et al.^40^ and Yong et al.^34^, where LightGBM consistently outperformed other standalone ML models with an RMSE of 0.08–0.58 mm/day and 0.041–0.315 mm/day, respectively. Further, there was a minor difference between LightGBM6 model and XGBoost6 model, with LightGBM6 having a little higher RRSE value (0.34–0.5%). It should be pointed out that while XGBoost had the highest KGE value among all models, our ranking also considered the low RMSE and RSSE values, which placed XGBoost in the second position.

**Table 10:** Average statistical indicators values for different standalone and hybrid ML models across studied stations during testing phase with various input combinations.

Model	Inputs combination	Gharb					Loukkos				
		KGE	*R* ^2^	RMSE	RRSE	Rank	KGE	*R* ^2^	RMSE	RRSE	Rank
RF1	T_max_, T_min_, R_s_	0.882	0.841	0.026	18.807	29	0.953	0.952	0.034	9.593	28
RF2	T_max_, T_min_, RH_mean_	0.762	0.652	0.068	29.891	46	0.730	0.612	0.098	27.187	46
RF3	T_max_, T_min_, R_s_, U_2_	0.913	0.945	0.021	12.211	15	0.942	0.961	0.031	8.682	17
RF4	T_max_, T_min_, RH_mean_, U_2_	0.832	0.801	0.054	20.591	38	0.786	0.723	0.082	22.947	36
RF5	T_max_, T_min_, RH_mean_, R_s_	0.912	0.913	0.025	14.936	22	0.938	0.958	0.033	9.093	22
RF6	T_max_, T_min_, RH_mean_, R_s_, U_2_	**0.919**	**0.966**	**0.020**	**10.066**	7	**0.933**	**0.966**	**0.030**	**8.296**	13
M5P1	T_max_, T_min_, R_s_	0.731	0.797	0.041	24.662	40	0.920	0.941	0.042	11.536	32
M5P2	T_max_, T_min_, RH_mean_	0.629	0.635	0.074	35.360	48	0.557	0.610	0.108	30.175	48
M5P3	T_max_, T_min_, R_s_, U_2_	0.927	0.911	0.024	14.801	21	0.951	0.952	0.035	9.650	29
M5P4	T_max_, T_min_, RH_mean_, U_2_	0.808	0.779	0.058	22.586	39	0.721	0.697	0.086	24.067	39
M5P5	T_max_, T_min_, RH_mean_, R_s_	0.889	0.916	0.024	15.739	24	0.931	0.952	0.036	9.999	31
M5P6	T_max_, T_min_, RH_mean_, R_s_, U_2_	**0.933**	**0.955**	**0.022**	**11.272**	12	**0.960**	**0.958**	**0.032**	**8.939**	20
XGBoost1	T_max_, T_min_, R_s_	0.900	0.833	0.027	19.749	34	0.966	0.948	0.036	9.978	30
XGBoost2	T_max_, T_min_, RH_mean_	0.769	0.632	0.070	31.163	47	0.732	0.585	0.102	28.406	47
XGBoost3	T_max_, T_min_, R_s_, U_2_	0.969	0.950	0.019	11.361	13	0.978	0.964	0.029	8.204	12
XGBoost4	T_max_, T_min_, RH_mean_, U_2_	0.873	0.801	0.054	19.976	36	0.807	0.691	0.088	24.405	40
XGBoost5	T_max_, T_min_, RH_mean_, R_s_	0.945	0.907	0.024	15.214	23	0.966	0.957	0.033	9.047	21
XGBoost6	T_max_, T_min_, RH_mean_, R_s_, U_2_	**0.982**	**0.975**	**0.017**	**8.253**	4	**0.977**	**0.969**	**0.027**	**7.630**	5
LightGBM1	T_max_, T_min_, R_s_	0.892	0.842	0.026	18.668	28	0.969	0.954	0.034	9.418	25
LightGBM2	T_max_, T_min_, RH_mean_	0.772	0.661	0.067	29.437	43	0.739	0.616	0.097	27.097	45
LightGBM3	T_max_, T_min_, R_s_, U_2_	0.967	0.950	0.019	11.248	11	0.980	0.968	0.028	7.774	9
LightGBM4	T_max_, T_min_, RH_mean_, U_2_	0.864	0.812	0.053	19.561	33	0.806	0.718	0.083	23.171	38
LightGBM5	T_max_, T_min_, RH_mean_, R_s_	0.944	0.918	0.023	14.212	18	0.973	0.964	0.030	8.337	15
LightGBM6	T_max_, T_min_, RH_mean_, R_s_, U_2_	**0.975**	**0.977**	**0.016**	**7.916**	3	**0.982**	**0.973**	**0.026**	**7.134**	3
RF-M5P1	T_max_, T_min_, R_s_	0.843	0.846	0.027	18.928	30	0.944	0.955	0.034	9.447	26
RF-M5P2	T_max_, T_min_, RH_mean_	0.734	0.669	0.067	29.657	44	0.687	0.631	0.096	26.724	41
RF-M5P3	T_max_, T_min_, R_s_, U_2_	0.915	0.948	0.019	11.851	14	0.943	0.964	0.030	8.349	14
RF-M5P4	T_max_, T_min_, RH_mean_, U_2_	0.825	0.811	0.053	20.097	37	0.766	0.735	0.081	22.509	33
RF-M5P5	T_max_, T_min_, RH_mean_, R_s_	0.907	0.922	0.023	14.258	19	0.936	0.962	0.031	8.759	19
RF-M5P6	T_max_, T_min_, RH_mean_, R_s_, U_2_	**0.920**	**0.973**	**0.018**	**9.211**	6	**0.940**	**0.970**	**0.028**	**7.755**	8
RF-LightGBM1	T_max_, T_min_, R_s_	0.890	0.847	0.025	18.302	25	0.967	0.956	0.033	9.218	23
RF-LightGBM2	T_max_, T_min_, RH_mean_	0.770	0.666	0.067	29.207	41	0.738	0.625	0.096	26.747	42
RF-LightGBM3	T_max_, T_min_, R_s_, U_2_	0.956	0.954	0.018	10.830	8	0.971	0.969	0.027	7.635	6
RF-LightGBM4	T_max_, T_min_, RH_mean_, U_2_	0.855	0.815	0.052	19.425	32	0.801	0.727	0.082	22.766	34
RF-LightGBM5	T_max_, T_min_, RH_mean_, R_s_	0.936	0.921	0.022	13.939	16	0.964	0.965	0.029	8.179	11
RF-LightGBM6	T_max_, T_min_, RH_mean_, R_s_, U_2_	**0.960**	**0.978**	**0.015**	**7.813**	2	**0.968**	**0.975**	**0.025**	**6.925**	1
RF-XGBoost1	T_max_, T_min_, R_s_	0.890	0.844	0.025	18.570	27	0.958	0.953	0.034	9.459	27
RF-XGBoost2	T_max_, T_min_, RH_mean_	0.767	0.656	0.067	29.759	45	0.733	0.615	0.097	27.089	44
RF-XGBoost3	T_max_, T_min_, R_s_, U_2_	0.931	0.953	0.019	11.216	10	0.954	0.965	0.029	8.157	10
RF-XGBoost4	T_max_, T_min_, RH_mean_, U_2_	0.845	0.809	0.053	19.832	35	0.794	0.725	0.082	22.855	35
RF-XGBoost5	T_max_, T_min_, RH_mean_, R_s_	0.924	0.917	0.023	14.461	20	0.946	0.961	0.031	8.746	18
RF-XGBoost6	T_max_, T_min_, RH_mean_, R_s_, U_2_	**0.938**	**0.974**	**0.017**	**8.685**	5	**0.946**	**0.971**	**0.027**	**7.619**	4
XGBoost-LightGBM1	T_max_, T_min_, R_s_	0.898	0.847	0.025	18.418	26	0.970	0.955	0.034	9.342	24
XGBoost-LightGBM2	T_max_, T_min_, RH_mean_	0.774	0.663	0.067	29.418	42	0.739	0.618	0.097	27.020	43
XGBoost-LightGBM3	T_max_, T_min_, R_s_, U_2_	0.968	0.954	0.018	10.831	9	0.980	0.969	0.028	7.686	7
XGBoost-LightGBM4	T_max_, T_min_, RH_mean_, U_2_	0.867	0.816	0.052	19.188	31	0.808	0.720	0.083	23.083	37
XGBoost-LightGBM5	T_max_, T_min_, RH_mean_, R_s_	0.946	0.918	0.022	14.131	17	0.971	0.963	0.030	8.351	16
XGBoost-LightGBM6	T_max_, T_min_, RH_mean_, R_s_, U_2_	**0.977**	**0.979**	**0.015**	**7.598**	1	**0.981**	**0.974**	**0.025**	**7.038**	2

Significant values are in bold.

Significant values are in bold underline.

In both study area, the worst performance were obtained by M5P2 < XGBoost2 < RF2 < LightGBM2, where on average KGE < 0.772, R^2^ < 0.661, RMSE > 0.067 mm/day and RRSE > 27.097% (Table 10). These findings contradict those of Granata^38^, who claimed that the M5P models performed well while the RF models were the least accurate. From Fig. 6, it can be observed that RF demonstrated strong performance during the training phase across all stations, likely due to its ensemble nature and robustness to noise.

However, LightGBM’s performance was often inferior to that of the RF and XGBoost models. This could be due to its different optimization approach and less effective capturing of complex relationships. This finding aligns with the study by Chia et al.^71^, which reported that LightGBM exhibited relatively weaker performance than RF and the M5 tree model during the training phase. The authors explained that LightGBM’s leaf-wise optimization required sufficient training data for effective performance^71^. Interestingly, the situation reversed during the testing phase, demonstrating that LightGBM performed better when properly trained. It is noteworthy to mention that for all ML model, the model’s performance difference was triggered by a difference in the training and testing datasets, such as temporal differences in meteorological data patterns during both phases.

In term of hybrid ML models, the Gharb’s performance ranking was XGBoost-LightGBM6 > RF-LightGBM6 > RF-XGBoost6 > RF-M5P6. The R^2^ (RRSE) values were 0.979 (7.598), 0.978 (7.813), 0.974 (8.685) and 0.973 (9.211) respectively. For Loukkos, the ranking models were XGBoost-LightGBM6 > RF-LightGBM6 > RF-XGBoost6 > RF-LightGBM3, with R^2^ (RRSE) values of 0.975 (6.925), 0.974 (7.038), 0.971 (7.619) and 0.969 (7.635) respectively. From Table 10,, the result shows that XGBoost-LightGBM perform the best. This might be because XGBoost is recognized for its regularization approaches and successful handling of complicated relationships, whereas LightGBM excels in efficient computation and handling huge datasets. Consequently, their hybridization could detect a wider range of patterns and improve overall performance. In contrast, the lowest performance occurred in RF-M5P2 < RF-LightGBM2 < XGBoost-LightGBM2 < RF-XGBoost2. Nonetheless, during training phase, RF-XGBoost model gave good performance, as shown in Fig. 6.

When comparing standoalone ML models with hybrid ML models, it was found that XGBoost-LightGBM was highly close to LightGBM in term of all statistical performance indicator. For instance, compared with XGBoost-LightGBM6, RRSE of LightGBM6, XGBoost6 and RF6 was in difference of 0.318% (0.096%), 0.655% (0.592%), 2.468% (1.258%), respectively for Gharb (Loukkos). This suggests that while the hybridization approach slightly improved model performance, the improvement was not significant. It’s worth noting that factors such as computational efficiency and implementation flexibility might impact the selection between standalone ML models and hybrid models. Therefore, LightGBM and XGBoost are the recommended ML model for estimating RET in our research study.

Collectively, these studies highlight the effectiveness of standalone and hybrid machine learning models for improved accuracy in RET estimation^33,34,38,40,51,54–56,71^. Similary, our findings indicate that standalone ML models exhibited better performance and accuracy compared to the empirical models for estimating daily RET at Gharb and Loukkos stations, using T_max_, T_min_, RH_mean_, R_s_, U_2_ as input variables.

## Research’s limitations

This research has some notable limitations that need to be addressed. The study’s period is relatively limited, spanning from 2011 to 2017. Hence, expanding the study’s period would provide a more comprehensive understanding of the ML models’ performance across diverse climatic fluctuations over a longer period. Moreover, the investigation of current study limited from subhumid to semi-arid climatic conditions only, further investigation should incorporate more climatic conditions to examine climatic variability thoroughly. Additionally, the accuracy and performance of ML and hybrid models heavily relied on the availability and quality of meteorological data, which could impact their effectiveness in areas with limited or incomplete data. To address this issue, future studies could utilise reanalysis data products, such as ERA5 ERA5-Land, and MERRA-2, which provide continuous, high-resolution data across extended temporal and spatial scales. Such data sources could prove invaluable in regions where in situ measurements are scarce. Moreover, ML models lack physical mechanisms, making it challenging to comprehend their inner workings and create accurate models without knowledge of functional specifications^74^. The issue of over-fitting and under-fitting during the training/testing phases of ML models, due to the dataset random division, may also affect model accuracy. Employing advanced validation techniques could help enhance model reliability and generalizability.

## Conclusion

Estimating reference evapotranspiration (RET) accurately has remained a major focus across a wide range of applications, including water resource management, agricultural water requirements, irrigation scheduling, and climate change assessments. In this research, we investigated the ability of four machine learning (ML) models, and their hybrid models along with eight empirical models (grouped in mass transfer-based, temperature-based, radiation-based and combination models) to estimate daily RET in subhumid and semi-arid irrigated perimeters in Morocco. For six input combinations, RF, M5P, XGBoost, LightGBM, RF-M5P, RF-XGBoost, RF-LightGBM, and XGBoost-LightGBM were all thoroughly evaluated. The results showed that combination models (particularly, Valiantzas 2013 (VAL2013b)) were the best empirical models and that temperature-based models generally outperformed radiation-based models. Compared with empirical models, ML models gave more accurate RET estimation, and the hybrid XGBoost-LightGBM models provided the highest statistical indicator values (KGE, R^2^, RMSE, RRSE). Interestingly, the standalone ML model LightGBM also demonstrated acceptable accuracy across all stations and input combinations, indicating its potential as a promising model for RET estimation with limited data. Moreover, the XGBoost model is also an intriguing alternative ML model. Overall, models with the input variables T_max_, T_min_, RH_mean_, R_s_, and U_2_ performed better for daily RET estimation.

The current research highlights the ML and hybrid models’ efficiency in estimating daily RET within two irrigated Moroccan perimeters. Ultimately, further investigations could explore additional ML algorithms, hybrid model configurations, and their relevance to long-term datasets at different time scales and various climate regions.

## Data Availability

The datasets used and/or analyzed during the current study are available from the corresponding author on reasonable request.

## References

[1] Satpathi, A. et al. Estimation of crop evapotranspiration using statistical and machine learning techniques with limited meteorological data: A case study in Udham Singh Nagar, India. *Theor. Appl. Climatol*. 10.1007/s00704-024-04953-3 (2024).

[2] Vishwakarma, D. K. et al. Methods to estimate evapotranspiration in humid and subtropical climate conditions. *Agric. Water Manag*. **261**, 107378 (2022).

[3] Mirzania, E., Vishwakarma, D. K., Bui, Q. A. T., Band, S. S. & Dehghani, R. A novel hybrid AIG-SVR model for estimating daily reference evapotranspiration. *Arab. J. Geosci.* **16**, 301 (2023).

[4] Dingman, S. L. *Physical Hydrology* (Waveland, 2015).

[5] Wanniarachchi, S. & Sarukkalige, R. A. Review on evapotranspiration estimation in agricultural water management: Past, present, and future. *Hydrology* **9**, 123 (2022).

[6] Jerin, J. N., Islam, A. R. M. T., Al Mamun, M. A., Mozahid, M. N. & Ibrahim, S. M. Climate change effects on potential evapotranspiration in Bangladesh. *Arab. J. Geosci.* **14** (2021).

[7] Dinpashoh, Y., Jahanbakhsh-Asl, S., Rasouli, A. A., Foroughi, M. & Singh, V. P. Impact of climate change on potential evapotranspiration (case study: west and NW of Iran). *Theor. Appl. Climatol*. **136**, 185–201 (2019).

[8] Haider, S. et al. Simulation of the potential impacts of projected climate and land use change on runoff under CMIP6 scenarios. *Water* **15**, 3421 (2023).

[9] Nouri, M. Drought assessment using gridded data sources in data-poor areas with different aridity conditions. *Water Resour. Manag*. **37**, 4327–4343 (2023).

[10] Noguera, I., Domínguez-Castro, F. & Vicente-Serrano, S. M. Flash drought response to precipitation and atmospheric evaporative demand in Spain. *Atmosphere (Basel)*. **12**, 165 (2021).

[11] Herold, N., Kala, J. & Alexander, L. V. The influence of soil moisture deficits on Australian heatwaves. *Environ. Res. Lett.* **11**, 064003 (2016).

[12] Raza, A. et al. Misconceptions of reference and potential evapotranspiration: A PRISMA-guided comprehensive review. *Hydrology* **9**, 153 (2022).

[13] Monteith, J. L. *Evaporation and Environment the State and Movement of Water in Living Organisms.* In *Symp. 19 Soc. Exp. Bid* (ed. Fogg, G.E.) (Cambridge University Press, 1965).

[14] Allen, R. G., Pereira, L. S., Raes, D. & Smith, M. *Crop Evapotranspiration: Guidelines for Computing Crop Water Requirements. Irrigation and Drainage Paper No 56. Food and Agriculture Organization of the United Nations (FAO), Rome, Italy*. 10.3390/agronomy9100614 (1998).

[15] Allen, R. G. et al. *The ASCE Standardized Reference Evapotranspiration Equation* (2005).

[16] Er-Raki, S. et al. Assessment of reference evapotranspiration methods in semi-arid regions: Can weather forecast data be used as alternate of ground meteorological parameters? *J. Arid Environ.* **74**, 1587–1596 (2010).

[17] Hamed, M. M., Khan, N., Muhammad, M. K. I. & Shahid, S. Ranking of empirical evapotranspiration models in different climate zones of Pakistan. *Land* **11**, 2168 (2022).

[18] Almorox, J., Quej, V. H. & Martí, P. Global performance ranking of temperature-based approaches for evapotranspiration estimation considering Köppen climate classes. *J. Hydrol.* **528**, 514–522 (2015).

[19] Valipour, M. Retracted: Comparative evaluation of radiation-based methods for estimation of potential evapotranspiration. *J. Hydrol. Eng.* **20**, 4014068 (2015).

[20] Zeggaf, T. A., El Mourid, M., Karrou, M. & Steduto, P. Comparaison des méthodes d’estimation de l’évapotranspiration de référence dans la région du Tadla-Maroc. *AL AWAMIA*. **100**, 73–84 (1999).

[21] Dai, L. et al. Comparison of fourteen reference evapotranspiration models with lysimeter measurements at a site in the humid Alpine Meadow, northeastern Qinghai-Tibetan Plateau. *Front. Plant. Sci.* **13** (2022). 10.3389/fpls.2022.854196 PMC9094065 35574067

[22] Bouhlassa, S. & Paré, S. Évapotranspiration De référence dans la région aride de tafilalet Au sud-est du Maroc. *Afr. J. Environ. Assess. Manag*. **11**, 1–16 (2006).

[23] Hadria, R., Benabdelouhab, T., Lionboui, H. & Salhi, A. Comparative assessment of different reference evapotranspiration models towards a fit calibration for arid and semi-arid areas. *J. Arid Environ.* **184**, 104318 (2021). 33082611 10.1016/j.jaridenv.2020.104318 PMC7522060

[24] Liou, Y. A. & Kar, S. K. Evapotranspiration estimation with remote sensing and various surface energy balance algorithms—A review. *Energies* **7**, 2821–2849 (2014).

[25] Elfarkh, J. et al. Evapotranspiration estimates in a traditional irrigated area in semi-arid Mediterranean. Comparison of four remote sensing-based models. *Agric. Water Manag*. **270**, 107728 (2022).

[26] El-Rawy, M. et al. An Integrated GIS and machine-learning technique for groundwater quality assessment and prediction in southern Saudi Arabia. *Water* **15**, 2448 (2023).

[27] Alshehri, F. & Rahman, A. Coupling machine and deep learning with explainable Artificial intelligence for improving prediction of groundwater quality and decision-making in Arid Region, Saudi Arabia. *Water* **15**, 2298 (2023).

[28] Abd El-Hamid, H. T. & Alshehri, F. Integrated remote sensing data and machine learning for drought prediction in eastern Saudi Arabia. *J. Coast Conserv.* **27**, 48 (2023).

[29] Nhu, V. H. et al. GIS-based gully erosion susceptibility mapping: A comparison of computational ensemble data mining models. *Appl. Sci.* **10**. (2039). 10.3390/app10062039 (2020).

[30] Prodhan, F. A., Zhang, J., Hasan, S. S., Sharma, P., Mohana, H. P. & T. P. & A review of machine learning methods for drought hazard monitoring and forecasting: Current research trends, challenges, and future research directions. *Environ. Model. Softw.* **149**, 105327 (2022).

[31] Pham, Q. B. et al. Groundwater level prediction using machine learning algorithms in a drought-prone area. *Neural Comput. Appl.* **34**, 10751–10773 (2022).

[32] Raza, A. et al. Performance evaluation of five machine learning algorithms for estimating reference evapotranspiration in an arid climate. *Water* **15**, 3822 (2023).

[33] Goyal, P., Kumar, S. & Sharda, R. A review of the artificial intelligence (AI) based techniques for estimating reference evapotranspiration: current trends and future perspectives. *Comput. Electron. Agric.* **209**, 107836 (2023).

[34] Yong, S. L. S., Ng, J. L., Huang, Y. F. & Ang, C. K. Estimation of reference crop evapotranspiration with three different machine learning models and limited meteorological variables. *Agronomy* **13**, 1048 (2023).

[35] Kisi, O. The potential of different ANN techniques in evapotranspiration modelling. *Hydrol. Process.* **22**, 2449–2460 (2008).

[36] Nourani, V., Elkiran, G. & Abdullahi, J. Multi-station artificial intelligence based ensemble modeling of reference evapotranspiration using pan evaporation measurements. *J. Hydrol.* **577**, 123958 (2019).

[37] Singh, A. K. et al. An integrated statistical-machine learning approach for runoff prediction. *Sustainability* **14**, 8209 (2022).

[38] Granata, F. Evapotranspiration evaluation models based on machine learning algorithms—A comparative study. *Agric. Water Manag*. **217**, 303–315 (2019).

[39] Vishwakarma, D. K. et al. Pre- and post-dam river water temperature alteration prediction using advanced machine learning models. *Environ. Sci. Pollut Res.*10.1007/s11356-022-21596-x (2022). 10.1007/s11356-022-21596-x PMC9244425 35763134

[40] Fan, J. et al. Light gradient boosting machine: An efficient soft computing model for estimating daily reference evapotranspiration with local and external meteorological data. *Agric. Water Manag*. **225**, 105758 (2019).

[41] Elbeltagi, A. et al. Prediction of meteorological drought and standardized precipitation index based on the random forest (RF), random tree (RT), and Gaussian process regression (GPR) models. *Environ. Sci. Pollut Res.* **30**, 43183–43202 (2023). 10.1007/s11356-023-25221-3 36648725

[42] Achite, M. et al. Performance of machine learning techniques for meteorological drought forecasting in the Wadi Mina Basin, Algeria. *Water* **15**, 765 (2023).

[43] Kumar, D. et al. Multi-ahead electrical conductivity forecasting of surface water based on machine learning algorithms. *Appl. Water Sci.* **13**, 192 (2023).

[44] Elbeltagi, A. et al. Forecasting long-series daily reference evapotranspiration based on best subset regression and machine learning in Egypt. *Water* **15**, 1149 (2023).

[45] Kushwaha, N. L. et al. Data intelligence model and meta-heuristic algorithms-based pan evaporation modelling in two different agro-climatic zones: A case study from Northern India. *Atmosphere (Basel)* **12**, 1654 (2021).

[46] Fan, J. et al. Evaluation of SVM, ELM and four tree-based ensemble models for predicting daily reference evapotranspiration using limited meteorological data in different climates of China. *Agric. Meteorol.* **263**, 225–241 (2018).

[47] Torsoni, G. B. et al. Soybean yield prediction by machine learning and climate. *Theor. Appl. Climatol*. **151**, 1709–1725 (2023).

[48] Masood, A. et al. Improving PM2.5 prediction in New Delhi using a hybrid extreme learning machine coupled with snake optimization algorithm. *Sci. Rep.* **13**, 21057 (2023). 38030733 10.1038/s41598-023-47492-z PMC10687010

[49] Shahhosseini, M., Hu, G., Huber, I. & Archontoulis, S. V. Coupling machine learning and crop modeling improves crop yield prediction in the US Corn Belt. *Sci. Rep.* **11**, 1606 (2021). 33452349 10.1038/s41598-020-80820-1 PMC7810832

[50] González, S., García, S., Del Ser, J., Rokach, L. & Herrera, F. A practical tutorial on bagging and boosting based ensembles for machine learning: Algorithms, software tools, performance study, practical perspectives and opportunities. *Inf. Fusion*. **64**, 205–237 (2020).

[51] Lachgar, N., Berrajaa, A., Essabbar, M. & Saikouk, H. Machine learning approach for reference evapotranspiration estimation in the Region of Fes, Morocco. In *International Conference on Digital Technologies and Applications*. 105–113 (Springer, 2023).

[52] Nagalla, R., Pothuganti, P. & Pawar, D. S. Analyzing gap acceptance behavior at unsignalized intersections using support vector machines, decision tree and random forests. *Proc. Comput. Sci.* **109**, 474–481 (2017).

[53] Pal, M. & Mather, P. M. An assessment of the effectiveness of decision tree methods for land cover classification. *Remote Sens. Environ.* **86**, 554–565 (2003).

[54] Elbeltagi, A. et al. Data intelligence and hybrid metaheuristic algorithms-based estimation of reference evapotranspiration. *Appl. Water Sci.* **12**, 152 (2022).

[55] El Hachimi, C., Salwa, B., Saïd, K. & Abdelghani, C. Early estimation of daily reference evapotranspiration using machine learning techniques for efficient management of irrigation water. *J. Phys. Conf. Ser.* **2224**, 12006 (IOP Publishing, 2022).

[56] Hachimi, C. et al. Smart weather data management based on artificial intelligence and big data analytics for precision agriculture. *Agriculture* **13**, 95 (2022).

[57] Van Buuren, S. & Groothuis-Oudshoorn, K. Mice: Multivariate imputation by chained equations in R. *J. Stat. Softw.* **45**, 1–67 (2011).

[58] Acharki, S., Amharref, M., El Halimi, R. & Bernoussi, A. S. Évaluation par approche statistique de l’impact des changements climatiques sur les ressources en eau: Application Au périmètre Du Gharb (Maroc). *Rev. Des. Sci. l’Eau/J. Water Sci.* **32**, 291–315 (2019).

[59] Dalton, J. Experimental essays on the constitution of mixed gases; on the force of stream or vapor from water and other liquids, both in a Torricellian vacuum and in air; on evaporation; and on the expansion of gases by heat. *Proc. Manch. Lit. Philos. Soc.* **5**, 536–602 (1802).

[60] Trabert, W. Neue Beobachtungen über Verdampfungsgeschwindigkeiten. *Meteorol. Z.* **13**, 261–263 (1896).

[61] Hargreaves, G. H. Moisture availability and crop production. *Trans. ASAE*. **18**, 980–984 (1975).

[62] Hargreaves, G. H. & Samani, Z. A. Reference crop evapotranspiration from temperature. *Appl. Eng. Agric.* **1**, 96–99 (1985).

[63] Allen, R. G. & Pruitt, W. O. Rational use of the FAO Blaney-Criddle formula. *J. Irrig. Drain. Eng.* **112**, 139–155 (1986).

[64] Irmak, S. & Haman, D. Z. Evapotranspiration: Potential or reference? *Agric. Eng. Fla. Coop. Ext. Serv. Inst. Food Agric. Sci. Univ. Fla. US ABE*. **343**, 1–3 (2003).

[65] Valiantzas, J. D. Simple: ET 0 forms of Penman’s equation without wind and/or humidity data. II: comparisons with reduced set-FAO and other methodologies. *J. Irrig. Drain. Eng.* **139**, 9–19 (2013).

[66] Breiman, L. Random forests. *Mach. Learn.* **45**, 5–32 (2001).

[67] Quinlan, J. R. Learning with continuous classes. In *5th Australian Joint Conference on Artificial Intelligence*. Vol. 92. 343–348 (World Scientific, 1992).

[68] Solomatine, D. P. & Xue, Y. M5 model trees and neural networks: Application to flood forecasting in the Upper Reach of the Huai River in China. *J. Hydrol. Eng.* **9**, 491–501 (2004).

[69] Chen, T. et al. Xgboost: Extreme Gradient Boosting. *R Package Version 0.4-2*. Vol. 1. 1–4 (2015).

[70] Ke, G., Ye, Q., Chen, W., Liu, T. Y. & LightGBM. *Highly Effic. Gradient Boost Decis. Tree* **30** (2016).

[71] Chia, M. Y., Huang, Y. F., Koo, C. H. & Fung, K. F. Recent advances in evapotranspiration estimation using artificial intelligence approaches with a focus on hybridization techniques—A review. *Agronomy* **10**. 10.3390/agronomy10010101 (2020).

[72] Raza, A. et al. Use of gene expression programming to predict reference evapotranspiration in different climatic conditions. *Appl. Water Sci.* **14**, 152 (2024).

[73] Raza, A. et al. Modelling reference evapotranspiration using principal component analysis and machine learning methods under different climatic environments. *Irrig. Drain.* **72**, 945–970 (2023).

[74] Wang, J. et al. Development of monthly reference evapotranspiration machine learning models and mapping of Pakistan—A comparative study. *Water* **14**, 1666 (2022).

[75] Grace, B. & Quick, B. A comparison of methods for the calculation of potential evapotranspiration under the windy semi-arid conditions of southern Alberta. *Can. Water Resour. J.* **13**, 9–19 (1988).

[76] Pandey, P. K., Dabral, P. P. & Pandey, V. Evaluation of reference evapotranspiration methods for the northeastern region of India. *Int. Soil. Water Conserv. Res.* **4**, 52–63 (2016).

[77] Kisi, O. Comparison of different empirical methods for estimating daily reference evapotranspiration in Mediterranean climate. *J. Irrig. Drain. Eng.* **140**, 4013002 (2014).

[78] Brutsaert, W. & Chen, D. Diurnal variation of surface fluxes during thorough drying (or severe drought) of natural Prairie. *Water Resour. Res.* **32**, 2013–2019 (1996).

[79] Irmak, S., Allen, R. G. & Whitty, E. B. Daily grass and Alfalfa-reference evapotranspiration estimates and alfalfa-to-grass evapotranspiration ratios in Florida. *J. Irrig. Drain. Eng.* **129**, 360–370 (2003).

[80] Arellano, M. G. & Irmak, S. Reference (potential) evapotranspiration. I: Comparison of temperature, radiation, and combination-based energy balance equations in humid, subhumid, arid, semiarid, and Mediterranean-type climates. *J. Irrig. Drain. Eng.* **142**, 4015065 (2016).

[81] Allen, R. G., Pereira, L. S., Raes, D. & Smith, M. Crop evapotranspiration-guidelines for computing crop water requirements-FAO Irrigation and drainage paper 56. *Fao Rome*. **300**, D05109 (1998).

[82] Droogers, P. & Allen, R. G. Estimating reference evapotranspiration under inaccurate data conditions. *Irrig. Drain. Syst.* **16**, 33–45 (2002).

[83] Raza, A. et al. Comparative assessment of reference evapotranspiration estimation using conventional method and machine learning algorithms in four climatic regions. *Pure Appl. Geophys.* **177**, 4479–4508 (2020).

[84] Raza, A. et al. Application of non-conventional soft computing approaches for estimation of reference evapotranspiration in various climatic regions. *Theor. Appl. Climatol*. **139**, 1459–1477 (2020).

[85] Raza, A. et al. Comparative study of powerful predictive modeling techniques for modeling monthly reference evapotranspiration in various climatic regions. *Fresenius Environ. Bull.* **30**, 7490–7513 (2021).

[86] Nouri, M., Ebrahimipak, N. A. & Hosseini, S. N. Estimating reference evapotranspiration for water-limited windy areas under data scarcity. *Theor. Appl. Climatol*. **150**, 593–611 (2022).

[87] Pelosi, A., Terribile, F., D’Urso, G. & Chirico, G. Comparison of ERA5-Land and UERRA MESCAN-SURFEX reanalysis data with spatially interpolated weather observations for the regional assessment of reference evapotranspiration. *Water* **12**, 1669 (2020).

[88] Allen, R. G. et al. Conditioning point and gridded weather data under aridity conditions for calculation of reference evapotranspiration. *Agric. Water Manag*. **245**, 106531 (2021).

